# Programmable Attenuation of Antigenic Sensitivity for a Nanobody-Based EGFR Chimeric Antigen Receptor Through Hinge Domain Truncation

**DOI:** 10.3389/fimmu.2022.864868

**Published:** 2022-07-22

**Authors:** Scott McComb, Tina Nguyen, Alex Shepherd, Kevin A. Henry, Darin Bloemberg, Anne Marcil, Susanne Maclean, Ahmed Zafer, Rénald Gilbert, Christine Gadoury, Robert A. Pon, Traian Sulea, Qin Zhu, Risini D. Weeratna

**Affiliations:** ^1^ Human Health Therapeutics Research Centre, National Research Council, Ottawa, ON, Canada; ^2^ Centre for Infection, Immunity and Inflammation, University of Ottawa, Ottawa, ON, Canada; ^3^ Department of Biochemistry, Microbiology, and Immunology, University of Ottawa, Ottawa, ON, Canada; ^4^ Department of Bioengineering, McGill University, Montréal, QC, Canada; ^5^ Institute of Parasitology, McGill University, Sainte-Anne-de-Bellevue, QC, Canada

**Keywords:** cellular immunotherapy, EGFR, CAR optimization, CAR-T, hinge domain, cancer selectivity, cell therapy

## Abstract

Epidermal growth factor family receptor (EGFR) is commonly overexpressed in many solid tumors and an attractive target for chimeric antigen receptor (CAR)-T therapy, but as EGFR is also expressed at lower levels in healthy tissues a therapeutic strategy must balance antigenic responsiveness against the risk of on-target off-tumor toxicity. Herein, we identify several camelid single-domain antibodies (also known as nanobodies) that are effective EGFR targeting moieties for CARs (EGFR-sdCARs) with very strong reactivity to EGFR-high and EGFR-low target cells. As a strategy to attenuate their potent antigenic sensitivity, we performed progressive truncation of the human CD8 hinge commonly used as a spacer domain in many CAR constructs. Single amino acid hinge-domain truncation progressively decreased both EGFR-sdCAR-Jurkat cell binding to EGFR-expressing targets and expression of the CD69 activation marker. Attenuated signaling in hinge-truncated EGFR-sdCAR constructs increased selectivity for antigen-dense EGFR-overexpressing cells over an EGFR-low tumor cell line or healthy donor derived EGFR-positive fibroblasts. We also provide evidence that epitope location is critical for determining hinge-domain requirement for CARs, as hinge truncation similarly decreased antigenic sensitivity of a membrane-proximal epitope targeting HER2-CAR but not a membrane-distal EGFRvIII-specific CAR. Hinge-modified EGFR-sdCAR cells showed clear functional attenuation in Jurkat-CAR-T cells and primary human CAR-T cells from multiple donors *in vitro* and *in vivo*. Overall, these results indicate that hinge length tuning provides a programmable strategy for throttling antigenic sensitivity in CARs targeting membrane-proximal epitopes, and could be employed for CAR-optimization and improved tumor selectivity.

## Introduction

Following the remarkable clinical success of CD19-targeted chimeric antigen receptor (CAR) therapies for the treatment of B-cell malignancies, design and development of novel CARs to treat common solid tumors is a highly active area of research and development (1). The first step for CAR development is the identification of an antigen binding domain (ABD) which is functional within a CAR; most typically this domain is composed of an antibody single-chain variable fragment (scFv) (2, 3). Previous work has also demonstrated that camelid single-domain antibodies (sdAbs), also called V_H_Hs or nanobodies, can also be used as effective CAR ABDs (4, 5), and a sdAb-based BCMA-targeting CAR-T; ciltacabtagene autoleucel (CARVYKTI™) has now gained FDA regulatory approval for treatment of relapsed or refractory multiple myeloma (6, 7). After identification of a functional ABD, molecular optimization can be used to further fine-tune the therapeutic properties of a CAR through alteration of various elements of the CAR molecule including the ABD, hinge, transmembrane domain, and intracellular signaling domains, each of which has been demonstrated to significantly impact the signaling properties and functionality of CARs (2, 3).

For some CARs, it may be desirable to attenuate the magnitude of the signal produced through CAR binding with the target antigen, resulting in lower antigen sensitivity/responsiveness. Such attenuated CARs can provide a more balanced level of signaling to favour long-term T cell persistence and/or to limit the reactivity to off-tumor antigen expression in normal tissues (8). Recent work has shown that decreased CAR signaling in CD19-specific CARs can be achieved by lowering the affinity of the ABD (9), altering the hinge or transmembrane domains (10–12), or engineering signaling domains with reduced activity (13). Intriguingly, these diverse molecular strategies have each been shown to enhance CAR-T persistence and therapeutic benefit in animal models, and shown some promise in at least one clinical trial thus far (9). These observations suggest that an optimal level of signaling may be somewhat lower than that of current generation clinical CD19-targeting CAR-T therapies. Thus, molecular optimization of CARs has emerged as a viable strategy for widening the therapeutic window of novel CAR-T therapies.

Epidermal growth factor receptor (EGFR) is one of the most commonly altered oncogenes in solid cancers, either through a variety of activating mutations or through over-expression of the native receptor (14, 15). EGFR has a relatively large extracellular domain with four subdomains (16), and is a well-established target for monoclonal antibodies and small-molecule inhibitors (17, 18). EGFR has also been explored as a target for CAR-T therapy, and clinical trials have been undertaken using ABDs specific for either the WT (19, 20) or a mutated tumor-specific form of the receptor known as EGFRvIII (21, 22). Recent clinical reports using EGFR-targeted CAR-T therapies in lung, biliary, and pancreatic cancers revealed no unmanageable toxicities and documented partial responses in some patients (23–26). EGFR is also under investigation as a target for bi-specific immune engaging therapy (27).

Although EGFR is attractive as an overexpressed tumor target, it is also expressed in normal tissues, and thus any therapeutic strategy must consider the potential for on-target off-tumor toxicity. In pre-clinical CAR-T work, it has previously been shown that the use of lower affinity EGFR ABDs can improve CAR-T selectivity for overexpressing tumor cells over normal tissues (28). Herein we present new camelid single-domain antibody (sdAb) CARs (sdCARs) with high on-target activity against human EGFR *in vitro* and *in vivo* and demonstrate that truncation of the hinge domain can be used to fine-tune CAR antigenic sensitivity and enhance selectivity for antigen overexpressing tumors. The results presented here extend previously established work examining the CAR hinge domain, demonstrating that for CARs targeting epitopes proximal to the target cell membrane, hinge-truncation offers a powerful and precise means to throttle CAR antigenic sensitivity. Such a strategy could provide a useful tool for optimizing CAR selectivity for tumor-associated antigens, elucidating CAR biology, and developing more complex multi-antigen targeting CAR therapeutics.

## Results

### High-Affinity EGFR-Specific sdAbs Are Effective Targeting Agents for EGFR-Specific CARs

**Figure 1:**
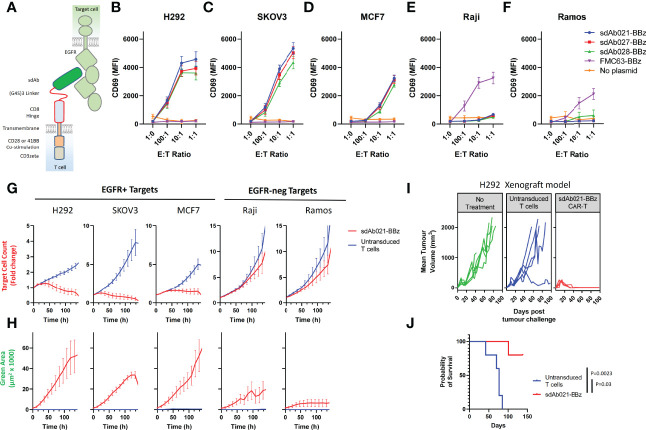
Identification of EGFR-specific sdAbs with CAR functionality. **(A)** The structural elements of human EGFR and anti EGFR-sdAb CAR tested in this study are shown. Three EGFR-specific sdAbs were cloned into a modular CAR backbone with 41BB and CD3z signaling domain *via* golden gate cloning. Jurkat cells were then electroporated with the resulting constructs. Control cells with no plasmid or with a CD19 (FMC63) scFv CAR were also tested here. Jurkat cells (30 000/well) transiently expressing various CAR plasmids as shown were co-cultured with varying doses of **(B, C)** EGFR-high H292 or SKOV3 cells, **(D)** EGFR-low MCF7 cells, **(E, F)** EGFR-negative Raji cells or Ramos Cells and examined for activation *via* staining with APC-labelled anti-human CD69 antibody. CAR-J results show the mean +/- SEM from a three independent experiments performed in duplicate. Primary human T cells were then transduced with lentivirus encoding the sdAb021-EGFR-sdCAR construct and tested for activity against mKate2-expressing target cells with varying EGFR expression. **(G)** Automated fluorescent counting was used to examine CAR-T mediated target cell killing and **(H)** expansion of EGFP-labelled CAR-T cells. Primary CAR-T results show the mean of 3 experiments performed in duplicate. NSG mice were injected with 6x10^6^ H292 human lung cancer cells subcutaneously, then treated with 5x10^6^ sdAb021-BBz CAR-T or untransduced control T cells intravenously (n=5 mice/group). **(I)** Growth of H292 tumors *via* regular caliper measurements is shown. **(J)** Probability of survival throughout the experiment is shown (P values are derived from a Mantel-Cox comparison of treatment groups).

Generation of camelid sdAbs against EGFR using DNA immunization and phage display was reported in a previous study (29) (see **Supplementary Figure 1** for an overview of the workflow). To assess whether these high-affinity EGFR sdAbs were functional in the context of a CAR we chose three sdAbs with varying affinities and epitopes on EGFR (**Table 1**) (29). EGFR-specific sdAbs were cloned into modular CAR plasmid backbones with a flexible linker-extended human CD8 hinge domain, CD28-transmembrane domain, either CD28 or 41BB co-stimulatory domain, and CD3zeta signaling domain (**Figure 1A**). The resulting EGFR-sdCARs were screened for responses to target cells with varying EGFR expression (**Supplementary Figures 2A, B**) using a high throughput CAR-Jurkat screening assay (32). Jurkat cells electroporated with any of three EGFR-specific sdCAR constructs showed specific upregulation of CD69 following co-culture with EGFR-high H292 or SKOV3 cells (**Figures 1B, C**), with lower but still obvious T-cell activation responses against EGFR-low MCF7 cells (**Figure 1D**). In contrast, EGFR-sdCAR molecules were not activated in response to EGFR-negative Raji or Ramos cells (**Figures 1E, F**), in contrast to CD19-specific CAR-T molecules used as a control. We also observed similar results with a CD28 based co-stimulatory domain containing CAR (**Supplementary Figure 2C**). These data demonstrate that high affinity EGFR sdCARs can induce strong responses to EGFR-expressing target cells, even those with very low apparent EGFR expression.

**Table 1:** Antigen-binding domains tested as CAR elements in this study.

Antibody Name	Type	Target	Affinity (*K*_D_)	Cross-Reactivity	Competition	Epitope Location	Ref
sdAb021	sdAb	EGFR (Erbb1)	38.5 nM	Human, Cyno	Competitive with sdAb027, partial with sdAb028	Unknown, likely domain IV	(29)
sdAb027	sdAb	EGFR (Erbb1)	1.6 nM	Human, Cyno	Competitive with sdAb021, partial with sdAb028	Unknown, likely domain IV	(29)
sdAb028	sdAb	EGFR (Erbb1)	9.0 nM	Human, Mouse	Partial competition with sdAb021 and sdAb027	Unknown, likely domain IV	(29)
Trastuzumab	scFv	HER2 (Erbb2)	n.d. (5 nM)^a^	Human, Cyno	Juxtamembrane conformational epitope in domain IV	Very membrane proximal	(30, 31)
F265	scFv	EGFRvIII	n.d. (27.5 nM)^a^	Unknown	Domain I/III neoepitope, non-competitive with F269	Membrane distal	(32) and unpublished
F269	scFv	EGFRvIII	n.d. (31.5 nM)^a^	Unknown	Domain I/III neoepitope of EGFR, non-competitive with F265	Membrane distal	(32) and unpublished

a Binding of scFvs was not evaluated. Monovalent affinity of full-length IgG is shown in parentheses.

Cyno, cynomolgus monkey.

To investigate whether these EGFR-sdCAR molecules have therapeutic potential, we next produced concentrated lentiviral vectors encoding one of the EGFR-sdCARs (sdAb021-BBz) with co-expression of an EGFP marker to identify CAR-expressing cells. We then transduced polyclonally activated PBMC-derived human donor T cells with EGFR-sdCAR lentivirus and expanded cells for 10 days. To assess EGFR-sdCAR functionality, CAR-T or control untransduced T cells were placed in low density co-culture with red-fluorescently labelled target cells with either high EGFR expression (H292 or SKOV3), low EGFR expression (MCF7), or no EGFR expression (Raji or Ramos). After overnight incubation EGFR-sdCAR cells showed clear upregulation of early T cell activation marker CD69 with EGFR+ target cells but not EGFR-negative target cells (**Supplementary Figure 2D**). We then examined the relative growth of CAR-T and various target cells for 7 days *via* automated fluorescence microscopy. Using automated cell counting to enumerate red target cells, clear target specific killing by EGFR-sdCAR-T cells but not corresponding untransduced T cells was observed (**Figure 1G** and **Supplementary Videos 1**–**3**). We did not observe similar killing of EGFR-negative Raji and Ramos cells (**Figure 1G**, **Supplementary Videos 5**–**6**). Similarly, enumeration of GFP+ CAR-T cells showed clear expansion in co-cultures with EGFR-positive but not EGFR-negative target cells (**Figure 1H** and **Supplementary Video 1**–**5**).

To confirm whether these EGFR-sdCAR molecules would have therapeutic activity *in vivo*, we performed a murine xenograft experiment wherein immunodeficient NOD-SCID-IL2Rγ-null (NSG) mice were implanted with 6M H292-lung cancer cells, followed by 5M CAR-T cells delivered intravenously 15 days after tumor injection. In this experiment 5/5 mice treated with EGFR-sdCAR cells showed complete tumor regression (**Figure 1I**) and significantly enhanced survival relative to those receiving untransduced T cells (**Figure 1J**). Overall, these results clearly demonstrate that these high affinity EGFR-sdAbs are functional CAR targeting moieties that result in CAR-T cells with high antigen sensitivity and *in vitro/in vivo* therapeutic activity.

### Hinge Truncation Can Be Used to Decrease EGFR-sdCAR Antigenic Sensitivity

**Figure 2:**
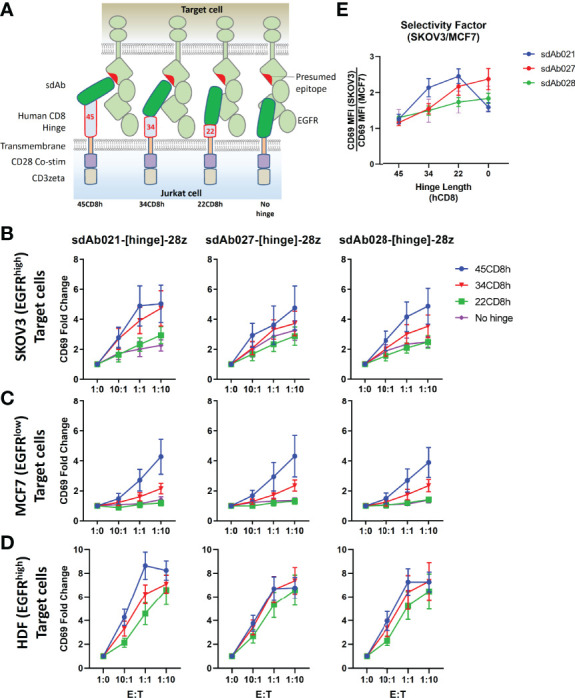
Hinge truncation decreases target response for EGFR-sdAb CAR constructs. **(A)** Structure of single domain antibody based CARs targeting human EGFR were generated with hinge domains of varying length [full length human CD8-hinge (45CD8h), truncated CD8-hinge (34CD8h or 22CD8h), or no hinge element. **(B, C)** Jurkat cells were electroporated with varying CAR plasmid constructs before co-incubation with no target cells (1:0 E:T), EGFR-high SKOV3 cells, or EGFR-low MCF7 cells. 30 000 CAR-Jurkat cells were incubated overnight with varying numbers of target cells before CAR/GFP+ cells were examined for activation *via* staining with APC-labelled anti-human CD69 antibody. **(D)** Shows the relative CAR-J activation of various constructs in response to SKOV3 or MCF7. Results show the mean +/- SEM from three experiments performed in duplicate.

Given the high sensitivity of EGFR-sdCAR T cells against even EGFR-low MCF7 target cells, we were concerned regarding possible on-target off-tumor effects. Thus we sought a controlled means by which we could attenuate the EGFR-sdCAR in order to enhance selectivity for EGFR-overexpressing cells. Based on previous work demonstrating that hinge domains of varying size derived from different human proteins can have a strong effect on CAR activity (33, 34), we hypothesized that progressive truncation of the hinge region could be used to more precisely attenuate target sensitivity. We cloned EGFR sdCAR constructs with either a full-length 45 amino acid human CD8 hinge (45CD8h) or N-terminally truncated CD8-hinge domains (34CD8h, 22CD8h, or no hinge; see **Figure 2A**). Jurkat cells transiently expressing the truncated hinge domain containing variants of all three EGFR-sdCARs showed progressively decreasing activation in response to EGFR-high SKOV3 target cells (**Figure 2B**). One possible explanation of the consistent effect of hinge truncation on antigenic sensitivity might be a reduction in surface expression for hinge-truncated CARs. Using a broadly reactive anti-sdAb antibody to label surface sdCAR expression we observed no impact of varying hinge domains on CAR surface expression (**Supplementary Figures 3A, B**). We also confirmed that EGFR sdCAR hinge truncation did not globally inhibit CAR-Jurkat responsiveness, as cells expressing hinge variant sdAb-CARs showed similar T-cell receptor-mediated responses to anti-CD3 antibody (**Supplementary Figures 3C, D**).

Next, we tested the response of EGFR sdCARs with truncated hinge domains against EGFR-low MCF7 cells, where we also observed decreased response with hinge-truncated EGFR-sdCARs (**Figure 2C**). Importantly, responses to EGFR-low MCF7 cells were more dramatically affected by hinge truncation than responses against EGFR-high SKOV3 cells. An analysis of hinge-modified CAR selectivity for SKOV3 vs. MCF7 cells shows the strongest selectivity enhancement with the sdAb021 sdCAR (**Figure 2D** and **Supplementary Figures 3E, F**), thus this construct was selected for further development. Overall, these results indicate that hinge truncation can be an effective tool to adjust the antigen-sensitivity of EGFR-sdCARs and enhance selectivity for EGFR-overexpressing cells.

### Single Amino Acid Hinge Truncation Provides Fine-Tuned Control of CAR-Antigen Response

**Figure 3:**
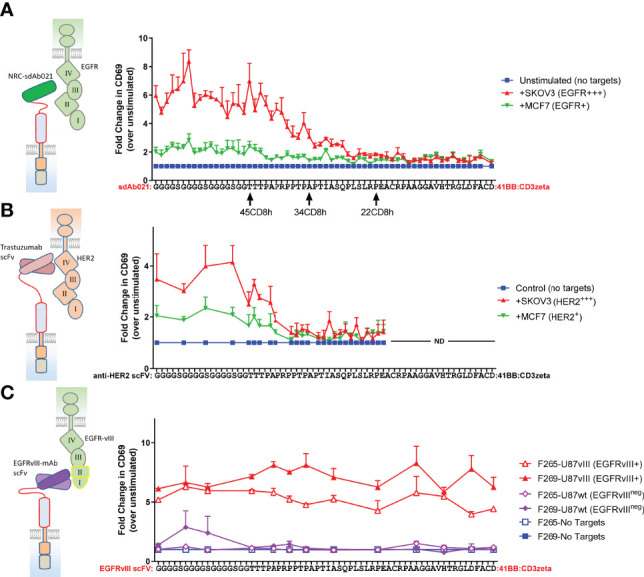
Single amino acid truncations can throttle CAR antigenic sensitivity for membrane proximal epitope targeting CARs. **(A)** A range of sdCAR constructs were produced containing EGFR-specific sdAb021 and N-terminally truncated versions of the linker-extended human CD8 hinge domains ranging between 1 and 62 residues in length. Jurkat cells were electroporated with the resulting constructs and then co-incubated at an effector to target ratio of 1:1 with EGFR-high SKOV3 cells or EGFR-low MCF7 cells. After overnight incubation, CAR/GFP+ cells were examined for activation *via* staining with APC-labelled anti-human CD69 antibody. **(B)** Similarly, HER2-specific trastuzumab-derivative scFv CAR constructs were generated with hinge domains of varying lengths. CAR-expressing Jurkat cells were co-incubated with HER2-high SKOV3 cells or HER2-low MCF7 cells and then CAR-T activation was assessed. **(C)** Similarly, EGFRvIII-specific CAR constructs were generated with hinge domains of varying lengths. CAR-expressing Jurkat cells were co-incubated with EGFRvIII-overexpressing U87vIII cells or EGFRvIII-negative U87wt cells and then CAR-T activation was assessed. All results show means +/- SEMs of three separate experiments.

Given our observations with truncated forms of the CD8-hinge domain, we wanted to more finely map the effects of progressive hinge truncation on an EGFR sdAb CAR. We also wondered whether hinge extension with an additional N-terminal flexible linker domain could be beneficial for antigen response. Thus, we designed an extended hinge domain with a 17 amino acids flexible linker appended to the N-terminus of the human CD8-hinge sequence [(GGGGS)_3_GG-45CD8h] within the EGFR-sdCAR construct. We generated an array of sdAb021-CAR constructs with N-terminal single amino acid deletions of the extended human CD8 hinge (every combination between 62 and 1 amino acid). Screening the EGFR-sdCAR single-residue hinge truncation library revealed a clear pattern of CAR activation (**Figure 3A**). CAR constructs containing a full human 45 AA CD8 hinge or longer produced strong responses to EGFR-high SKOV3 cells and lower but consistent responses to EGFR-low MCF7 cells. While there was some variation, the addition of a flexible linker extension to the CD8-hinge domain did not appear to have a consistent positive or negative effect on EGFR-sdCAR response. In this assay, EGFR-sdCARs with CD8 hinge sizes between 45 and 26 amino acids showed a progressive decrease in CAR activation, while CAR constructs with hinges 26 or less amino acids within their hinge domains showed no response to EGFR-expressing targets over CAR-J cells in the absence of targets (**Figure 3A**). Taken together, these data suggest that hinge truncation can provide precise control over EGFR-sdCAR antigenic sensitivity.

### Epitope Location Is a Critical Determinant of CAR Hinge Sensitivity

According to current understanding, the location of the CAR target epitope should be critical in determining the minimal hinge size needed for CAR antigenic responsiveness (35). We do not have definitive data as to the epitope(s) targeted by the EGFR sdAbs used in our sdCAR constructs, though cross-reactivity analysis is suggestive of binding to the membrane proximal domain IV of EGFR (29) (**Table 1**). Thus, to more carefully investigate the role of epitope location in CAR hinge dependence, we generated smaller arrays of truncated hinge CARs with known target epitopes. We selected scFv-CARs based on either trastuzumab, which is known to bind a highly membrane proximal epitope of HER2 (30), or EGFRvIII-specific antibodies we have previously reported to show activity in CAR format (32), which by necessity must bind the membrane distal neo-epitope of EGFRvIII (**Table 1**). The membrane-proximal targeting trastuzumab-derivative scFv-CAR required a very long hinge element; with HER2 scFv CARs losing activity rapidly when hinge domains shorter than a full CD8-hinge were used (**Figure 3B**). In contrast, membrane-distal targeting EGFRvIII CARs maintained full activation with all hinge formats, even when the entire CD8 hinge domain was deleted (**Figure 3C**). These data support a model wherein epitope location is a critical determinant of whether a hinge domain is required for CAR functionality, and thus hinge truncation can only be effectively employed for fine-tuning antigenic sensitivity in membrane-proximal targeting CAR constructs.

### Hinge-Truncation Progressively Diminished CAR Cell Binding to Target Cells

**Figure 4:**
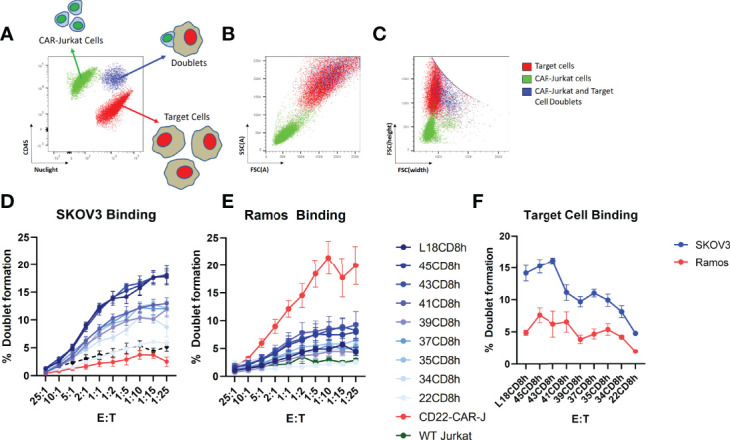
Hinge truncation progressively diminishes CAR-cell binding with target cells. Jurkat cells with stable expression of sdAb021 EGFRsdCAR were generated through lentiviral transduction and cell sorted for similar surface expression. EGFR-sdCAR-Jurkat cells were then mixed at varying doses with mKate2-expressing target cells and co-incubated for 30 minutes at 37°C. **(A)** Flow cytometry was used to assess the number of CAR-Jurkat cells, target cells, and CAR-Jurkat/target doublets. **(B)** Examining the size/granularity parameter shows that doublet cells (blue) appear similar in size to the larger target cell population. **(C)** Examining the height/width parameter indicates that identifies the doublet population as wider than the target cells alone (blue vs red). The proportion of CAR-Jurkat cells engaged in doublet formation with **(D)** EGFR-high SKOV3 cells or **(E)** EGFR-negative Ramos cells were quantified using gating as described in the main text. **(F)** The target cell binding across varying hinge constructs at a fixed effector:target ratio of 1:5 is shown. Graphs show the mean +/- SEM from three experiments performed in duplicate.

We next wished to investigate how hinge truncation alters cell-cell interaction between CAR-T cells and target cells. Thus, we generated Jurkat cell lines stably expressing EGFR-targeted CAR constructs with varying hinge sizes and performed cell sorting to isolate pools of EGFR-sdCAR with similar CAR expression level. EGFR-sdCAR cells were then stained with anti-CD45 and mixed at varying effector to target ratios with red-fluorescently labelled EGFR-high SKOV3 cells or EGFR-negative Ramos cells. Co-cultures were then incubated for 30 minutes before using flow cytometry to quantitate the number of CAR-Jurkat cells, target cells, and CAR-target doublets (**Figure 4A**). Examination of cell size and height/width ratio support an interpretation that CAR-target doublets can be quantified *via* this method (**Figures 4B, C** and **Supplementary Figure 4D**). We observed progressively decreasing target cell binding for EGFR-sdCAR cells with truncation of CAR hinge domains (**Figure 4D**). EGFR-sdCAR cells with a hinge length of 22CD8h showed similar binding to control WT-Jurkat cells or CD22-targeted CAR-Jurkat cells. In contrast to SKOV3 binding, interaction with EGFR-negative Ramos cells remained low for all EGFR-sdCAR cells, but was high for a control CD22-targeted CAR (**Figure 4E**). Examining the level of target binding at a fixed ratio across hinge variants tested reveals a clear pattern of decreased target cell binding with hinge truncation (**Figure 4F**). These results indicate that shortening the hinge domain directly limits the ability of EGFR-sdCAR cells to bind to antigen expressing target cells.

### Hinge-Truncation Progressively Diminishes CAR Functionality in Primary CAR-T Cells *In Vitro*

**Figure 5:**
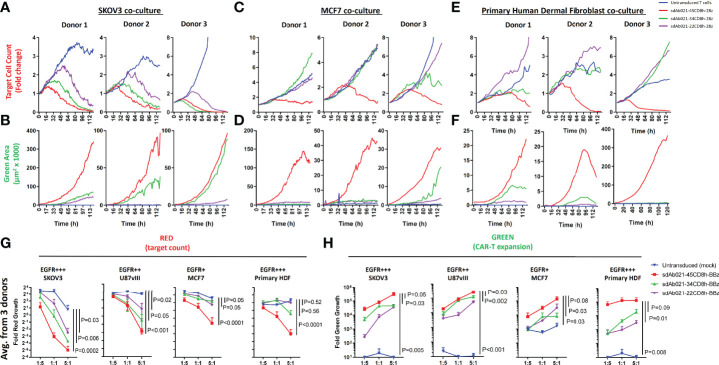
Hinge truncation progressively diminishes tumor cell killing and expansion of primary sdCAR-T cells in response to EGFR expressing target cells. Concentrated lentiviral particles encoding hinge-modified EGFR-specific sdAb021 CARs as well as a GFP marker were generated. Peripheral blood T cells were isolated from 3 independent healthy human donors before polyclonal expansion and lentiviral transduction. Varying doses of sdCAR-T cells or mock transduced cells (empty CAR backbone lentivirus) were placed at an E:T ratio of 1:1 in low density co-culture (2000 sdCAR-T cells and 2000 target cells). Co-cultures were examined over 7 days *via* live fluorescence microscopy (Incucyte) to differentiate red-fluorescent (NLS-mKate2) target cell counts or total area of green-fluorescent (NLS-NanoGreen) CAR-T cells. **(A, B)** Depicts the response to EGFR-high SKOV3 targets, **(C, D)** depicts the response to EGFR-low MCF7 targets, **(E, F)** depicts the response to EGFR-high healthy donor human dermal fibroblast cells. Each graph depicts automated cell counts or fluorescent areas from a single independent experiment. **(G)** Day 5 mean fold change in various target cell growth at varying E:T ratio for hinge-variant EGFR-sdCAR-T cells derived from 3 donors is shown +/- SEM, P values are derived from a two-way ANOVA comparison of response curves for untransduced T cell co-cultures with CAR-T cells expressing hinge-truncated constructs. **(H)** Similarly the mean EGFR-sdCAR-T fold expansion at day 5 of hinge-variant CAR co-cultures from 3 donors is shown +/-SEM is shown. P values are derived from a two-way ANOVA comparison on 45CD8h-hinge containing constructs with other constructs tested in parallel.

We next undertook extensive experiments to confirm whether the attenuation of antigenic sensitivity for hinge-truncated EGFR-sdCAR molecules observed in Jurkat cells functioned similarly in primary CAR-T cells. Thus, EGFR-sdCAR-T cells were generated from blood T cells from three healthy human donors using CAR lentivirus encoding three hinge-variant forms of sdAb021-41BB-CD3z sdCAR. As observed in Jurkat cells, CAR-T surface labelling using an anti-single domain antibody did not reveal any effect of hinge truncation on CAR surface expression (**Supplementary Figure 5**). Primary EGFR-sdCAR-T:tumor cell co-cultures were then monitored for tumor cell (red fluorescence) and CAR-T cell (green fluorescence) growth over 7 days without media changes. Consistent with observations in Jurkat cells, hinge truncation progressively diminished the ability of EGFR-sdCAR-T cells to kill SKOV3 tumor cells (**Figure 5A**) and undergo CAR-T cell expansion (**Figure 5B** and **Supplementary Figure 6A**). Despite progressive attenuation of CAR responses, all hinge formats showed more SKOV3 killing and CAR-T expansion than untransduced cells. In contrast to this, only the full-length CD8-hinge domain EGFR-sdCAR-T cells (sdAb021-45CD8h-28z) showed consistent target killing and CAR-T expansion in response to EGFR-low MCF7 cells (**Figures 5C, D** and **Supplementary Figure 6B**). To further investigate the behavior of EGFR-sdCAR-T cells in the context of non-malignant EGFR-expressing cells, we performed a similar co-culture assay using HDF cells, which have a high level of EGFR expression (**Supplementary Figure 2**). Despite high EGFR expression, hinge truncation exerted a more potent effect on EGFR-sdCAR killing against HDF cells and CAR-T expansion, similar to that observed for MCF7 cells (**Figures 5E, F**). These results are consistent with many observations regarding T-cell inhibitory activity of fibroblast cells (36), furthermore these results underline importance of cellular context dependent nature of CAR-T target cell interaction.

To more robustly test EGFR-hinge variant CAR-T responses to target cells, we plated co-culture experiments of CAR-T cells derived from three donors at varying effector:target ratios with each of the three target cells discussed above. These results revealed a highly significant and consistent pattern of enhanced selectivity for EGFR-high targets (SKOV3 or U87vIII) over EGFR-low tumor targets (MCF7) or healthy-donor HDF cells with hinge-truncated EGFR-sdCARs (**Figures 5G, H**). Interestingly, expansion of EGFR-sdCAR-T cells with truncated hinge domains seemed to be somewhat less sensitive to hinge truncation than target killing (**Figure 5H**). Overall these results provided strong evidence that that hinge truncation can be used to attenuate antigenic sensitivity and re-balance CAR selectivity for EGFR-overexpressing tumors.

We also performed an investigation of the effect of hinge truncation on CAR-T killing over an extended period of time. We isolated hinge modified sdCAR-T cells after primary challenge with antigen-overexpressing SKOV3 cells using the low density co-culture assay as described above and re-challenged the sdCAR-T cells by re-plating with additional target cells (**Supplementary Figure 7**). Re-challenged cells maintained similar relative selectivity observed in primary challenge as determined by target killing and CAR-T expansion, which both decreased with hinge length. These results indicate that re-stimulated CAR-T cells show similar or higher antigenic discrimination as observed in primary challenge, with progressively decreasing antigen-induced expansion following serial challenge.

### Hinge-Truncated EGFR CAR-T Cells Selectively Kill SKOV3 Tumor Cells and Spare Healthy Donor Derived Cells in Triple Co-Cultures

**Figure 6:**
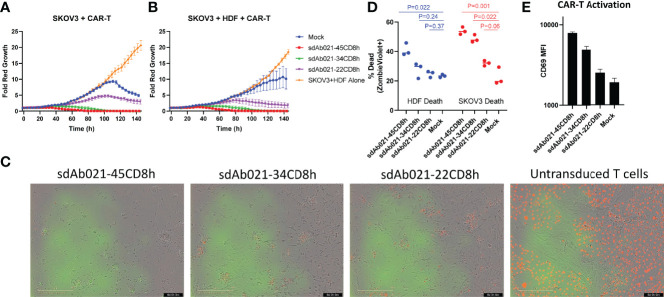
Hinge truncated CAR-T cells maintain enhanced tumor selectivity in triple co-cultures with both healthy donor cells and SKOV3 tumor cells. Hinge variant EGFR sdCAR cells were placed in co-culture with **(A)** equal number of mKate2-expressing SKOV3 cells or **(B)** equal numbers of SKOV3 and unmodified human healthy donor derived dermal fibroblasts (HDF). Graphs depict the average fold change in growth of red fluorescent tumor cells over time from CAR-T cells derived from 3 independent donors. **(C)** Pictures show the state of triple co-cultures after 6 days of incubation. In a similar experiment, triple co-cultures of hinge variant EGFR-sdCAR cells, HDF, and SKOV3 cells were incubated overnight before examining **(D)** HDF or SKOV3 viability and **(E)** T-cell activation marker CD69 expression *via* flow cytometry. Graphs depict the mean results for CAR-T or control T cells from 3 independent blood donors. P values indicate a student T-test comparison for variance between differing hinge constructs, an ANOVA comparison of survival curves is also shown where marked.

We next tested an *in vitro* model of tumor selectivity wherein both EGFR-overexpressing tumor cells and healthy donor derived HDFs were both present in triple co-culture. We plated hinge variant EGFR sdCAR-T cells and fluorescently labelled SKOV3 tumor cells with or without the addition of unmodified healthy donor HDFs (effector:target:HDF ratio of 1:1 or 1:1:1 respectively). Examining growth kinetics of SKOV3 cells specifically, the presence of HDF cells did not alter SKOV3 growth kinetics with or without CAR-T cells (**Figures 6A, B**, **Supplementary Figure 8A**). The addition of untransduced T cells to the HDF-SKOV3 co-culture slightly slowed SKOV3 growth, but we did not observe SKOV3 killing (**Figures 6A, B** and **Supplementary Video 6**). As observed in dual co-cultures, EGFR sdCAR-T cells mediated strong SKOV3 killing, which was similarly reduced with shorter hinge CAR constructs with or without HDF cells (**Figures 6A, B**). Visual examination of co-cultures revealed complete CAR-T mediated killing of SKOV3 and HDF cells with the long hinge sdAb021-45CD8h sdCAR construct, with progressive sparing of HDF cells in truncated hinge CARs (**Figure 6C**, **Supplementary Videos 7**–**9**). Results were similar across CAR-T cells derived from three independent blood donors (**Supplementary Figures 8A,B**). Varying E:T within triple co-cultures showed a similar pattern of CAR-T activity to that observed with SKOV3 and CAR-T cells (**Supplementary Figure 8C**).

In order to directly quantitate the relative survival of SKOV3, HDF, and CAR-T cells in this triple co-culture model, we performed a similar experiment wherein 1:1:1 mixture of CAR-T:HDF : SKOV3 from all three donors were cultured for 24 hours and then stained with ZombieViolet fixable viability dye, human CD45, and human CD25, to examine for relative target cell death and T cell activation simultaneously. Using human CD45 staining and red fluorescence to differentiate HDF, CAR-T, and SKOV3 cells (**Supplementary Figure 8D**), we observed preferential killing of SKOV3 cells across all EGFR sdCAR hinge formats (**Figure 6D**). In contrast to SKOV3, where increased target killing was observed for all CAR-T constructs, short hinge EGFR-sdCAR-T cells did not significantly increase HDF cell death relative to untransduced T cells (**Figure 6D**). We finally confirmed that T cell activation was steadily increased for longer-hinge EGFR sdCAR-T cells, as assessed by CAR-T CD69 expression (**Figure 6E**). Overall, these results demonstrate that hinge truncation increases tumor selectivity for EGFR sdCAR-T cells even in simultaneous co-culture with both healthy cells and cancer cells.

### *In Vivo* CAR-T Response Is Progressively Diminished With Hinge Truncation

**Figure 7:**
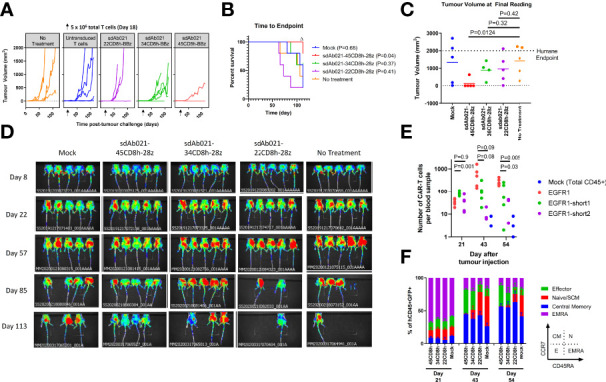
Hinge truncated EGFR sdCAR-T cells show progressively diminished response to human lung cancer xenografts *in vivo*. NOD/SCID/IL2γ-chain-null (NSG) mice were injected subcutaneously with 2×10^6^ SKOV3 cells stably expressing mKate2. Mice (N=5 mice/group) were injected with 5×10^6^ total T cells (~1x10^6^ CAR-T cells) intravenously. **(A)** Tumor volume was assessed using caliper measurements and **(B)** time to defined humane endpoint (tumor volume 2000 mm^3^) was assessed. P values are derived from Mantel-Cox test of survival curves in comparison with untreated mice. Δ Note that the experiment was ended early due to non-experimental related animal facility shutdown, and thus the final tumor measurement of day 112 is shown in **(C)**. **(D)** Fluorescence imaging was performed at varying timepoints after tumor cell injection. **(E)** Blood was also collected at selected timepoints after tumor challenge to quantify the proportion of human CD45+ cells that were GFP/CAR+. **(F)** Staining for human CCR7 and CD45RA was used to assess the differentiation status of CAR/GFP+ cells or total T cells for mice treated with mock-transduced CAR-T cells. See inset for gating strategy used to define effector, naïve/SCM, central memory, or effector memory-RA+ (EMRA) cells.

Finally, we wished to investigate whether our *in vitro* observations regarding the relationship between hinge length and CAR-T activity were consistent in an *in vivo* xenograft model. Using the relatively slow growing SKOV3 model, mice were challenged with 2×10^6^ SKOV3 tumor cells subcutaneously. After allowing 18 days for tumor implantation, all mice formed palpable tumors and were injected with 10 million hinge variant EGFR-sdCAR-T cells or untransduced CAR-T cells. There was a progressive increase in tumor growth in all mice with decreasing therapeutic activity with shorter hinge domain EGFR-sdCARs (**Figure 7A**). While the longest 45CD8-hinge CAR-T cells showed significantly increased survival, those mice treated with hinge-truncated sdCAR-T cells did not have a survival benefit (**Figure 7B**). The progressive effect of hinge truncation can be clearly observed in the final tumor volume measurement taken at day 112 post tumor challenge, although only those mice treated with the longest hinge format sdAb021-45CD8h-BBz showed a significant decrease in tumor volume (**Figure 7C**) which could also be observed by *in vivo* imaging of red-fluorescent tumor cells (**Figure 7D**). Examining CAR-T cells in the blood of treated mice revealed a consistent pattern of increased expansion of sdCAR-T cells with longer hinge regions at 43 and 54 days post-tumor injection (**Figure 7E**). Analysis of T cell differentiation revealed increased numbers of naïve or stem cell memory cells (N/SCM), and decreased effector sdCAR-T cell populations for hinge truncated EGFR-sdCARs (**Figure 7F**), supporting the interpretation that hinge truncation has a predictable and progressive effect on EGFR-sdCAR function *in vivo*.

**Figure 8:**
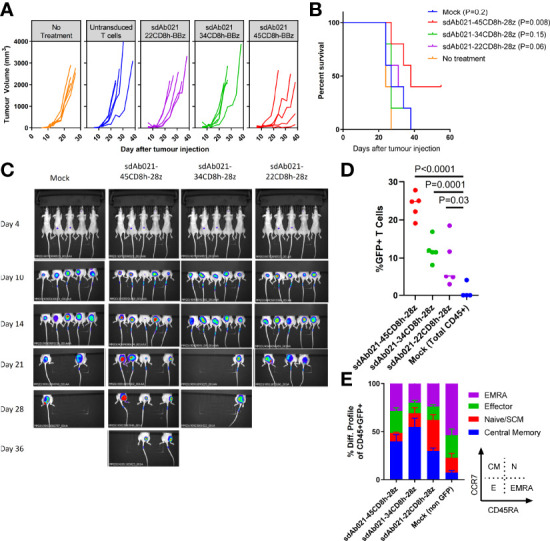
Hinge truncation results in similar progressively diminished EGFR-specific sdCAR response to human glioblastoma xenografts *in vivo*. **(A)** NSG mice were injected (N= 5 mice/group) subcutaneously with 1×10^6^ U87vIII cells stably expressing mKate2. At 7 and 14 days post-tumor challenge mice were injected intratumorally with 1×10^7^ total T cells (approx. 2.5×10^6^ hinge-modified sdCAR-T cells) or with untransduced control T cells (mock). Tumor growth was monitored *via* caliper measurements. N=5 mice per group. **(B)** Mice were sacrificed at pre-determined endpoints based on animal condition or tumor volume >2000 mm^3^. P values are derived from Mantel-Cox test of survival curves in comparison with untreated mice. **(C)** *In vivo* imaging was performed to examine the mKate2 fluorescent signal associated with tumor cells. **(D)** Blood was drawn from challenged mice and examined for the proportion of sdCAR-transduced cells (GFP+) within the hCD45+ lymphocyte fraction at the final timepoint where all experimental mice were alive (day 21). **(E)** sdCAR-T cells, or total hCD45+ cells for mock T cell treated mice, were examined for differentiation status using antibody staining for human CCR7 and CD45RA. See inset for gating strategy used to delineate effector, naïve/SCM, central memory, or effector memory-RA+ (EMRA) cells.

To expand on these experiments, we also performed a similar xenograft experiment in which wild-type or hinge modified sdCAR-T cells were delivered intratumorally in the relatively more aggressive EGFR-high U87-vIII glioblastoma xenograft model. We observed similar progressive decreases in anti-tumoral effect (**Figures 8A–C**) and CAR-T expansion with truncated hinge EGFR-sdCAR constructs (**Figure 8D**). Furthermore, we observed increased naïve/SCM CAR-T cell populations associated with hinge truncation (**Figure 8E**). Taken together, these results demonstrate that hinge truncation can be used for reprogramming antigenic sensitivity for this EGFR-sdCAR constructs *in vitro* and *in vivo.*

## Discussion

We sought here to develop a novel EGFR-specific CAR construct that can discriminate between cells with high level expression of EGFR and cells with lower EGFR expression, as found on many non-malignant cells in the body (37). Despite some variation in binding affinity for the three EGFR sdAb moieties tested here (1.6 to 38 nM; **Table 1**), all three sdCARs showed similar strong responses to both EGFR-high SKOV3 and EGFR-low MCF7 target cells. Interestingly, we note EGFR-sdCAR responsiveness to MCF7 cells, despite no apparent reactivity of the purified sdAbs to EGFR-low MCF7 cells which we previously reported (29). These results are consistent with previous observations that EGFR specific CARs are relatively insensitive to ABD affinity up to the micromolar range (28), and underscore the exquisite antigen sensitivity of CAR-T cells to respond to and lyse even very low antigen expressing target cells (38–40). This phenomenon possibly relates to the extreme multi-valency of both the sdCAR and EGFR on their respective cells; increased valency is well known to lead to significant avidity effects that boost the apparent affinity of biological interactions (41–44), and these would presumably apply to CAR-T cells as well. These results can be taken to indicate that lowering ABD affinity may be a somewhat blunt tool for modulation of CAR antigenic sensitivity.

In experiments presented here we did not observe significant differences in antigenic sensitivity for CARs using EGFR-targeting sdAb elements ranging in affinity between 1.6 and 38.5nM, regardless of the hinge domain used. Consistent with this, previous reports using affinity modulation to increase selectivity of CAR constructs for antigen-overexpressing cells required over 1000-fold change in target affinity (28). Based on this previous data and our experience here, it seems that affinity modulation of binding elements is a blunt instrument for modulation of CAR sensitivity and would also carry the risk of generating unpredictable binding behavior, such as unexpected off-target binding, elevated tonic signaling, or poor protein stability., Thus, we wished to pursue an alternate molecular strategy for down-modulating EGFR-sdCAR antigenic sensitivity.

The use of hinge domains derived from various antibody isotypes or receptor ectodomains has been well- documented to have powerful influence on antigenic responsiveness of particular CAR constructs (2), and the strategy to employ different spacer domains of varying length has also been previously explored (3, 33, 45, 46). To our knowledge however, this is the first report of a simple truncation strategy of the human CD8-hinge domain which is most commonly employed in CAR designs. We find that truncations of a human CD8 hinge by as little as a single amino acid can be a remarkably precise throttle mechanism for CAR antigenic sensitivity. For the EGFR sdCAR we tested most extensively here, there was a steady drop-off of antigenic CAR response over a range of 10 to 20 amino acids within the CD8 hinge motif. Our hinge-truncation data using either membrane proximal (trastuzumab) or membrane-distal (anti-EGFRvIII-mAbs) based CAR constructs provides a further molecular demonstration of how epitope location is a determinant of hinge-sensitivity for CAR molecules reacting to tumor cells.

Using Jurkat cells, we were able to demonstrate that truncation of the CAR hinge domain can progressively decrease the upregulation of the early activation marker CD69 following co-incubation with antigen-expressing target cells, but this data does not necessarily provide mechanistic insight into how hinge truncation would affect fundamental processes involved in CAR signaling. With this in mind, we also explored the most upstream process involved in CAR activity, that being binding interaction between CAR antigen binding domain and tumor cells. Thus, we employed a short term co-culture assay to examine the immediate effect of hinge truncation on CAR-target cell interaction, similar to previous work investigating T-cell binding to tumor cells (47). These experiments mirrored results with CD69, with those truncated hinge CAR molecules showing less binding to target cells, despite similar membrane expression levels. These results demonstrate that hinge truncation can be used to selectively limit the ability of CAR cells to bind antigen-expressing target cells. A deeper exploration of how hinge truncation can be used to reprogram more downstream processes involved in CAR signaling, such as the activation of important transcription factors (NFAT, NFκB, AP1) will be an important area for further study. However, since target interaction is the primary initiator of CAR-T signaling and downstream function; we believe that the evidence of our observations in regards to cell binding provide strong evidence that the strength of CAR binding and signaling can be fine-tuned *via* hinge length modulation.

In contrast to hinge truncation, we found that extension of the CD8 hinge domain does not have a strongly positive or negative effect on CAR response, at least as determined by the CAR-J assay. Similarly, membrane-distal epitope targeting EGFRvIII CARs showed similar responses across all hinge formats tested here. Previous work has indicated that longer hinges can decrease *in vivo* activity for membrane-distal epitope targeting CARs (45), but follow-up studies pinpointed the effect to be related to FC-binding by IgG-hinge motifs rather than hinge length specifically (46). It is also important to note that the multi-[GGGS] linker format used to extend our hinge is a highly flexible sequence typically employed for scFv engineering, and thus may allow for adequate ABD motility and close CAR packing unlike long hinge domains used in previous studies (33, 45). While it is clear that membrane-distal CAR molecules cannot function with a hinge domain which is too short, no firm conclusion on the impact of an excessively long hinge can yet be drawn.

Due to the relatively more demanding technical requirements of testing CAR-T constructs in primary T cells we only tested a limited number of CAR constructs within primary cell assays. Nonetheless, data presented here provide additional evidence that molecular optimization using transient CAR expression in Jurkat cells is predictive of responses in stably transduced primary CAR-T cells, as we have previously reported (32). The wider use of such optimization methodology could lead to improved ABD/hinge design for future CAR products. It may be possible for instance to design CARs with customized signaling for application in CD4, CD8, gamma-delta T cells, or NK cells.

Intriguingly, despite high expression of EGFR on healthy donor HDF cells, we observed obvious and significant decrease in *in vitro* HDF killing by hinge-truncated EGFR-sdCAR-T cells and increased selectivity for SKOV3 tumor cell killing. These results reflect the highly contextual nature of CAR-T activity, and underline that responses are never strictly dependent on CAR-antigen interaction alone. T-cell interaction with target cells involves a great number of cellular receptors, including many positive and negative regulators of T cell function (48), thus it is unsurprising that CAR-T cells should show differential selectivity towards primary versus malignant cells despite similar antigen expression. While it is not clear how specific these results are to HDF cells in particular, the consistency of our observations here clearly indicate that hinge-truncated CARs would have lower overall sensitivity to EGFR in all contexts and thus lower likelihood of on-target off-tumor toxicity.

For the *in vivo* models tested here, only the highest activity/longest hinge format was able to drive significant tumor regression and enhanced mouse survival, underlining the fundamental trade-off between maximizing on-target/on-tumor activity and minimizing on-target/off-tumor responses. It is not clear how predictive results from such xenograft tumor models are to human clinical efficacy, and thus the lack of efficacy results here should be taken with caution. Also, perhaps more extensive dose response *in vivo* studies would be required to demonstrate the differential activity of the hinge truncated CAR constructs. As murine xenograft models are not directly translatable to human clinical efficacy; such studies using large numbers of animals would not be ethically warranted. The data presented here does clearly demonstrate the ability to modulate the activity of the EGFR sdAb CAR with hinge length modulation. Translatability of these findings in human clinical setting would have to be evaluated with appropriately designed clinical trials.

The programmable antigenic sensitivity demonstrated here raises the possibility of a number of novel applications. For example, a clinical strategy wherein first-in-man trials could be performed with high safety/low activity truncated constructs with progressive hinge length escalation in subsequent dosing or patient cohorts may be possible. Alternatively, one could also envision dosing with a longer hinge CAR with an appropriate suicide switch for initial rapid tumor debulking followed by safer/low activity hinge truncated CAR for long term tumor control/surveillance. Finally, it may also be possible to incorporate such reduced sensitivity EGFR-sdCARs in multi-antigen targeting CAR strategy that will ultimately recognize and lyse tumor cells in more selective fashion. The data presented here provide strong evidence that generating similar CAR constructs with partially truncated hinge domains can be a powerful tool to engineer CAR molecules with desired antigenic sensitivity.

## Materials and Methods

### CAR Cloning

Three previously reported EGFR-specific sdAb sequences (29) were cloned into a modular CAR backbone using PCR amplification and single-pot restriction ligation as previously described (32). EGFR-sdCAR constructs bearing either a full-length human 45 amino acid CD8 hinge (45CD8h) or progressively N-terminally truncated hinge variants (34CD8h, 22CD8h, or no hinge) were cloned using Gibson assembly. A library of sdAb021-CAR truncation mutants with single amino acid N terminal truncations of the human CD8 hinge extended with an additional N-terminal flexible linker [(GGGGS)_3_GG-CD8h] was generated using a modular hinge-CAR with convenient type-IIs restriction sites integrated into the construct 3’ of the sdAb coding region. An array of DNA encoding truncated CD8 hinge domains of varying lengths (all possible variants between 60 and 1 amino acid) were synthesized as DNA fragments (Twist Bioscience, San Francisco, CA, USA) and cloned into the sdAb021-modular-hinge-BBz-GFP CAR construct using single-pot restriction ligation. Limited hinge truncation libraries with defined-target CARs were generated by exchanging the sdAb021 sequence with HER2 or EGFRvIII specific scFv sequences. Trastuzumab derived scFv sequences were generated based on previously reported mutant forms of trastuzumab with enhanced avidity for recognizition of HER2-overexpressing tumor cells (44), whereas EGFRvIII-targeting scFvs were generated as previously reported (32). Both HER2- and EGFRvIII-scFvs were in a VH-(G4S)_3_-VL format.

### Cell Lines and Culture

Other than U87MG and HDF cells, all others were obtained from American Tissue Culture Collection (ATCC, Manassas, VA, USA). The glioblastoma cell line U87 MG-WT and U87 MG-vIII (U87-vIII, expressing EGFRvIII *via* retroviral transduction and sorting) were kindly provided by Professor Cavnee from the Ludwig Institute for Cancer Research, University of California, San Diego (San Diego, CA, USA) (49). HDFs were purchased from Cell Applications (San Diego, CA, USA). The T-cell lines used were Jurkat, and target cells used were SKOV3, MCF7, U87 MG vIII, Raji, and Ramos. Target cells were transduced with lentivirus containing NucLight Red (Sartorius, Essen BioScience, Bohemia, NJ, USA), a third generation HIV-based, VSV-G pseudotyped lentivirus encoding a nuclear-localized mKate2. Nuclight positive cells were obtained by selection with puromycin. HDFs were cultured in all in one ready to use fibroblast growth medium (Cell Applications, Inc). U87 MG, SKOV3, MCF7, were cultured in DMEM supplemented with 10% fetal bovine serum (FBS), 2mM L-glutamine, 1mM sodium pyruvate and 100 µg/mL penicillin/streptomycin. Jurkat, Raji, and Ramos were cultured in RPMI supplemented with 10% FBS, 2mM L-glutamine, 1mM sodium pyruvate and 100 µg/mL penicillin/streptomycin. These cell lines were tested and found negative for the presence of mycoplasma contamination by PCR.

### CAR-J Assay

High-throughput assessments of CAR function were performed by CAR-J assay according to a previously outlined protocol (32). Briefly, 5×10^5^ cells were suspended in 100 µL of Buffer 1SM (5 mM KCl, 15 mM MgCl_2_, 120 mM Na_2_HPO_4_/NaH_2_PO_4_, 25 mM sodium succinate, and 25 mM mannitol; pH 7.2) and incubated with 2 µg of pSLCAR-CAR plasmids as described in the text or with no plasmid control. Cells and plasmid DNA in solution were transferred into 0.2 cm generic electroporation cuvettes (Biorad Gene Pulser; Bio-Rad, Hercules, CA, USA) and immediately electroporated using a Lonza Nucleofector I (Lonza, Basel, Switzerland) and program X-05 (X-005 on newer Nucleofector models). Cells were cultured in pre-warmed recovery media (RPMI containing 20% FBS, 1 mM sodium pyruvate and 2 mM L-glutamine) for 4 h before being co-cultured with EGFR-expressing target cells U87 MG-vIII, MCF7 and SKOV-3 or negative control Ramos and Raji cells. Electroporated Jurkat cells were added to varying numbers of target cells in round bottom 96-well plates in effector to target (E:T) ratios ranging from 1:10 to 100:1 (effector to target ratio) or with no target cells (or an E:T of 1:0) and cultured overnight before being staining with allophycocyanin (APC)-conjugated anti human-CD69 antibody (BD Biosciences #555533). Flow cytometry was performed using a BD- LSRFortessa (BD Biosciences, San Diego, Ca, USA) and data was analyzed using FlowJo software (FlowJo LLC, Ashland, OR, USA) and visualized using GraphPad Prism (GraphPad Software, Inc., San Diego, CA, USA, USA).

### Short Term CAR-Jurkat Target Cell Binding Assay

Jurkat cells stably expressing EGFR-sdCARs with varying hinge domains were generated using lentiviral transduction as described below, using an MOI of 1. CAR-expressing cells were then stained with anti-sdAb antibody described below and subjected to cell sorting using a MoFlo Astrios cell sorting flow cytometer (Beckman Coulter, Mississauga, ON, Canada) in order to isolate populations of EGFR-sdCAR-Jurkat cells with similar levels of CAR surface expression. Healthy cultures of EGFR-sdCAR-Jurkat cells were then stained with anti-human CD45 (BD Bioscience, USA, Cat#563717), then resuspended at 5x10^5^ cells per mL in RPMI-complete media. Healthy cultures of SKOV3 or Ramos target cells were then harvested and resuspended in fresh RPMI-Complete media at 5x10^5^ cells per mL. CAR-Jurkat and target cell cultures were then mixed at varying effector to target ratios in a 96-well plate, followed by incubation at 37°C for 30 minutes and immediate assessment *via* flow cytometry using a BD LSR Fortessa device. The proportion of CAR-target cell doublet formation was assessed as described in the main text. Experiments were repeated 3 times in duplicate.

### Plate-Bound Anti-CD3 CAR-J Stimulation Assay

Stable Jurkat cells expressing hinge variant sdAb021-BBz CARs were generated using lentiviral transduction similarly as described below, with cell sorting of GFP+ cells to generate stable CAR-expressing cell lines. OKT-3 stock (BioLegend Inc, San Diego, CA, USA) was diluted to 50 µg/mL, then diluted serially threefold. Plates were left at 4°C for 24 hours to bind. Unbound antibody was washed off the plate using PBS. 10^4^ stable Jurkat cells expressing hinge-variant CARs were added to each well; in parallel hinge variant Jurkat-CAR cells were combined with varying doses for SKOV-3 target cells. Plates were incubated at 37°C 5% CO_2_ for 24 hours before staining with mouse anti-human CD69-APC and analysis *via* flow cytometry.

### Human Peripheral Blood Mononuclear Cell (PBMC) Isolation

Heparinized whole blood was collected from healthy donors under informed consent by venipuncture and transported at room temperature from Ottawa Hospital Research Institute. Blood was diluted 1:1 with Hank’s balanced salt solution (HBSS) and PBMCs were isolated by Ficoll-Paque™ density gradient centrifugation. Briefly, samples layered on Ficoll-Paque™ gradient were centrifuged for 20 min at 700 × g without applying a brake. The PBMC interface was carefully removed by pipetting and was washed twice with HBSS by stepwise centrifugation for 15 min at 300 × g. PBMCs were resuspended and counted by mixed 1:1 with Cellometer ViaStain™ acridine orange/propidium iodide (AOPI) staining solution and counted using a Nexcelom Cellometer Auto 2000 (Nexcelom BioScience, Lawrence, MA, USA). T cells from were then activated with Miltenyi MACS GMP T cell TransAct™ CD3/CD28 beads and seeded 1x10^6^ T cells/mL in serum-free StemCell Immunocult™-XF media (StemCell Technologies, Vancouver, Canada) with clinical grade 20 U/mL human IL-2 (Novartis, Basel, Switzerland).

### Human Primary T Transduction by Spinfection

High concentration lentiviral particles encoding various sdCAR constructs were generated as previously described (32). After 24 h of T cell stimulation with beads, T cells were transduced with sdCAR-GFP lentiviral vectors (multiplicity of infection = 10) by spinfection. Briefly, lentivirus was added to T cells (1×10^6^ cells/mL) and the mixture was centrifuged at 850 × g for 2 h at 32°C. After centrifugation, cells were incubated at 37°C for another 2 h. After incubation, cells were plated in a 24 well plate (100,000 cells/mL/well in a total of 1.5 mL) in StemCell Immunocult™-XF supplemented with 20 U/mL IL-2. Media with IL-2 was added at 48 and 72 h post transduction to promote CAR-T cell proliferation without disrupting the cells. Cell number and viability were assessed by AOPI staining and counting using a Nexcelom Cellometer. CAR-T cells were propagated until harvest on days 7, 9, 14, and 21 to assess the efficiency of transduction and to characterize T cell subpopulations by flow cytometry. CAR-T cells that had returned to a resting state (as determined by decreased growth kinetics, day 10 post-T cell activation) were used for assays.

### Continuous Live-Cell Imaging Cytotoxicity Assay

Cytotoxicity of the CAR-T cells was assayed using a Sartorius IncuCyte^®^ S3 (Essen Bioscience). Tumor cells, U87-MG-vIII-NucLight, MCF7-NucLight, and SKOV3-NucLight or HDF-NucLight, Raji-NucLight, and H292-Nuclight were resuspended in StemCell ImmunoCult™-XF with 20 U/mL IL-2 and plated in a flat bottom 96-well plate (2000 cells/well). CAR-T cells or control T cells were added into each well in a final volume of 200 µL per well in StemCell ImmunoCult™-XF with 20 U/mL IL-2 at varying effector:target ratios and co-cultured for 7 days at 37°C. In triple co-culture experiments, unmodified HDF and SKOV3-Nuclight cells were first plated at 1000 cells/well each, then additional CAR-T cells were added at varying doses as described in the text. Images were taken at regular intervals in light phase and under red (ex. 565-605 nm; em. 625-705 nm) or green fluorescence (ex. 440-480 nm; em. 504-544 nm). The assays were repeated thrice with T cells derived from independent blood donors. For one donor, CAR-T cells challenged once or twice with EGFR-high SKOV3 cells were rechallenged with various freshly plated target cells after 7 day of co-culture. Automated cell counting of red (target) or green (CAR-T) cells was performed using IncuCyte^®^ analysis software and data were graphed using GraphPad Prism.

### Animal Studies

NOD/SCID/IL2Ry^-/-^ (NSG, JAX #005557) mice were purchased from Jackson Laboratories and maintained by the Animal Resource Group at the National Research Council of Canada. Eight-week-old NSG mice were injected with 2×10^6^ SKOV3-NucLight in 100 ul of Hanks’ Balanced Salt Solution (HBSS) subcutaneously. Eighteen days post tumor injection (when tumor reached approximately 5 mm × 5 mm), mice were retro-orbitally injected with 1×10^7^ fresh day 10 post-activation mock T cells or T cells transduced with various CAR-T cells as described in the text. Tumors were measured using calipers twice a week and mice were imaged *via* IVIS *in vivo* imager for red-fluorescence signal (expressed on tumor cells) once a week. For SKOV3 study, eight days after tumor cell injection, cryo-preserved CAR-T cells were thawed, washed with PBS, and 5×10 total T cells (with 20-25% CAR transduction) were immediately delivered intratumorally, ensuring equal distribution of tumor sizes between groups. For the alternative U87 MG vIII model experiments, mice were subcutaneously injected with 1×10^6^ fluorescently labelled U87 MG-vIII cells described above, a number we previously determined to consistently produce a palpable tumor within 7 days; mice were then treated at day 15 post-tumor challenge with 10^7^ total T cells (approximately 2-3M CAR-T cells). Tumor growth was evaluated three times per week using calipers by trained animal technicians blinded to specific treatment groups. Mice were also assessed for tumor growth using IVIS *in vivo* imaging to examine red fluorescence derived from the NLS-mKate2 marked U87-MG-vIII cells. In order to minimize requirement for animal shaving to the area immediately around the implanted tumor a blanket was used to obscure part of animals during imaging. Primary endpoint was tumor size above 2000 mm^3^, with secondary endpoints determined by overall animal health and well-being. Mice were euthanized when they met pre-specified endpoints. The study was approved by the NRC-HHT Institutional Animal Care Committee and was conducted in accordance with Canadian Council on Animal Care (CCAC) guidelines. Tumor growth and survival (humane endpoint) curves were generated using GraphPad Prism.

### Generation of a Monoclonal Anti-sdAb Antibody for Assessment of sdCAR Surface Expression

CAR expressing Jurkat cells or primary T cells were generated as indicated elsewhere and assessed for surface expression using an Alexa-Fluor647 labelled murine monoclonal anti-sdAb antibody generated in house. In brief, mice were immunized with sdAb fragments produced in *E. coli* followed by B cell isolation and hybridoma formation as previously reported (50). Clonal hybridoma supernatants were screened for reactivity against a number of plate bound sdAbs to identify broadly sdAb-reactive monoclonal antibodies. Antibodies were then sequenced and transferred to a recombinant murine IgG2a backbone for production in CHO cells. Antibodies were then purified *via* protein-G column before labelling with Alexa-Fluor 647 using a commercial kit. This reagent was confirmed to react broadly against sdAb-CAR expressing cells but not scFv-CAR expressing Jurkat cells. This reagent was then employed through cell staining and flow cytometric assessment as described in the text for quantification of relative sdCAR surface expression.

### Flow Cytometry and Antibodies

For *in vitro* studies, cells were stained with antibodies as indicated in the text, incubating the cells for 15 minutes at room temperature before washing with PBS. Cells were then resuspended in PBS and examined using a BD LSR Fortessa Flow cytometer. Staining of human EGFR was performed using anti-human EGFR-PE-CF594 (BD Biosciences, Cat #563431), and anti-sdCAR staining was performed using in house antibody generated as described above. For *in vitro* triple co-cultures were plated within 24-well plates with 50000 SKOV3 and HDF cells and varying CAR-T numbers as described in the text. After overnight incubation, cultures were stained with hCD45 and hCD25 antibodies, washed, stained with ZombieViolet Fixable viability dye (BioLegend USA, Cat #423113), followed by wash and fixation using 2% formalin in PBS. For *in vivo* experiments, blood was obtained from mice at various time points post CAR-T injection. Blood was washed with cold phosphate-buffered saline (PBS) and pelleted at 350 × g for 5 min at 4°C. Red blood cells were lysed using Red Blood Cell Lysing Buffer Hybri-Max (Sigma-Aldrich, St. Louis, MO, USA). Human T cells were identified and analyzed for activation/differentiation status using the following antibodies: hCD45-APC-H7, hCD45RA-BV650, hCD45RO-PE-CF594, hCD27-BUV737, hCCR7-PE, hCD4-BUV395, and hCD8-PerCP-Cy5.5 (all antibodies from BD Biosciences). CAR expression was measured indirectly *via* expression of GFP incorporated in CAR constructs. To evaluate exhaustion, staining by an hPD-1-BV421 antibody was evaluated. T cell activation was detected using hCD25-PE-Cy7 and hCD69-BV786 antibodies. For *in vivo* studies, a BV711-labeled antibody against mouse CD45 was used to identify murine cells.

## Data Availability Statement

The original contributions presented in the study are included in the article/**Supplementary Material**. Further inquiries can be directed to the corresponding author.

## Ethics Statement

Studies involving animals were reviewed and approved by NRC-HHT Institutional Animal Care Committee.

## Author Contributions

SMc, TN, AM, DB, KH, TS, RG, and RW contributed to conceptualization, experimental design, analysis, and interpretation of results; TN, AS, DB, SMa, CG, RP, AZ, and QZ contributed to technical development, performed experiments, and compiled data; SMc, TN, KH, and RW contributed to writing and editing the manuscript. All authors contributed to the article and approved the submitted version.

## Funding

This work was funded by the National Research Council Canada Disruptive Technology Solutions Cell and Gene Therapy challenge program.

## Conflict of Interest

The authors declare that the research was conducted in the absence of any commercial or financial relationships that could be construed as a potential conflict of interest.

## Publisher’s Note

All claims expressed in this article are solely those of the authors and do not necessarily represent those of their affiliated organizations, or those of the publisher, the editors and the reviewers. Any product that may be evaluated in this article, or claim that may be made by its manufacturer, is not guaranteed or endorsed by the publisher.

## Supplementary Material

Supplementary Figure 1 Workflow for discovery and optimization of novel tumor selective sdCAR constructs: Antigen preparation, llama immunization, phage library preparation and panning, cell binding, CAR cloning, high-throughput CAR screening, and fine-tuning of CAR signaling in Jurkat cells through hinge length modulation was performed for several novel EGFR sdCAR constructs. Subsequently, confirmatory experiments were performed to test lead sdCARs *in vitro* and *in vivo* using primary human CAR-T cells.

Supplementary Figure 2 EGFR-CAR-Jurkat and primary CAR-T cells can respond to EGFR-positive cells with a wide range of antigen expression. Jurkat, Raji, MCF7, U87vIII, SKOV3, and healthy donor derived primary human dermal fibroblast (HDF) cells were stained with varying amounts of commercial PE-CF594 anti-human EGFR antibody and examined by flow cytometry as indicated in **(A)**. **(B)** The mean fluorescence intensity of each cell type is shown. Results demonstrated lack of expression of EGFR in Jurkat and Raji, low expression in MCF7 cells, high expression in U87vIII, and very high expression for SKOV3and HDF cells. Histograms depict the fluorescence signal from a single experiment. **(C)** High throughput CAR-Jurkat screening was performed with CAR constructs composed of EGFR-sdAb binders, an extended hinge domain, CD28-transmembrane domain, CD28 co-stimulatory domain, and CD3zeta signaling domain. CAR-J results show the mean +/- SEM from three independent experiments performed in duplicate. **(D)** Human EGFR-sdCAR-T cells were generated as per description in methods section. CAR-T cells were then co-cultured at E:T of 1:1 with 300 000 cells CAR-T cells and 300 000 target cells with varying EGFR expression in a 96-well plate. After overnight co-incubation, cells were stained with human CD69-APC and examined *via* flow cytometry. Results show the mean of a single duplicate experiment for CAR-T cells from each independent donor.

Supplementary Figure 3 CAR Surface expression is not altered with hinge domain modification. **(A)** Jurkat cells were electroporated with various EGFR-sdCAR plasmids (GFP expressing) and incubated overnight then stained with an Alexa-Fluor647 labelled murine monoclonal anti-sdAb antibody to examine surface CAR expression. WT Jurkat cells (left panel) show no GFP and no binding to anti-sdAb, whereas all other EGFR-CARs show clear simultaneous GFP expression and binding to anti-sdAb. **(B)** Mean fluorescence intensity for AF647-anti-sdAb stained CAR-expressing Jurkat cells, gated on GFP expression is shown. **(C)** CAR-J CD69 expression of hinge modified EGFR sdCARs stimulated with plate bound OKT3 stimulation overnight are shown in comparison to **(D)** CAR-J cells stimulated with EGFR-high SKOV3 cells at varying dose. Results are representative of 3 repeated experiments and demonstrate modulation of antigen-specific response *via* the truncated hinge. **(E)** Data presented for CAR-J assay against EGFR-high SKOV3 cells as described in were reorganized here to show the activation of EGFR sdCARs over a range of hinge lengths at an effector to target ratio of 1:10, varying from a full 45 amino acid human CD8 hinge to no hinge. **(F)** Data from CAR-J data against EGFR-low MCF7 cells organized *via* hinge length are shown.

Supplementary Figure 4 Gating scheme for CAR-Jurkat target cell doublet formation assay Flow cytometry was performed to analyze the interaction of EGFR-sdCAR-Jurkat cells with various target cells as described in the main text. To analyze the proportion of CAR cells engaged in binding to target cells the following gating hierarchy was used: size/granularity gating to remove cell debris, followed by gating on total CD45+ population to isolate CAR-Jurkat cells, followed by gating on GFP+/mKate+ population to isolate CAR-Jurkat target cell doublets. Plots shown are representative of 3 repeated experiments performed in duplicate.

Supplementary Figure 5 CAR Surface expression in primary CAR-T cells is not changed with hinge length **(A)** Primary T cells from two independent donors were purified, expanded, and transduced with CAR lentivirus as described in the methods section. CAR-T cells were stained with AlexaFluor647 anti-sdAb antibody and examined *via* flow cytometry to assess CAR surface expression. GFP/CAR+ T cells show a clear binding to the sdAb antibody, not seen in untransduced Mock T cells. **(B, C)** Histograms and mean fluorescence intensity for anti-sdAb stained CAR-T cells is shown, with no consistent effect of hinge truncation on CAR surface expression.

Supplementary Figure 6 Hinge truncation results in reduced response to antigen-low and non-malignant EGFR+ target cells **(A, B)** Primary sdCAR-T cells were generated from two independent blood donors as described in the text and placed at low density co-cultures in a 96-well plate. Cells were then monitored for growth *via* live fluorescence microscopy over 7 days. Graphs display the total areas of GFP+ cells as enumerated through automated cell counting. **(C)** Representative images are provided for various sdCAR-T (GFP/green) or control mock T cells with target cell (mKate2/red) co-cultures 4 days after plating at an effector:target ratio of 5:1. 3 independent experiments were performed with CAR-T cells from independent donors as shown in the text.

Supplementary Figure 7 Hinge-truncated sdCAR-T cells maintain selectivity for EGFR overexpressing cells following re-challenge. Donor blood derived T cells were transduced with lentiviral particles encoding the EGFR-specific sdAb021 sdCAR with varying hinge domains as described in the text. The resulting sdCAR-T cells were challenged *via* co-culture with EGFR-high SKOV3 cells and examined for target cell expansion or **(D)** sdCAR-T cell expansion as described above. Challenged cells were then diluted 1/10 with fresh media and challenged with **(B, E)** EGFR-high SKOV3 cells, **(G, I)** EGFR-medium U87vIII cells, or **(K, M)** EGFR-low MCF7 cells. Similarly, cells challenged twice with SKOV3 cells were re-challenged with various target cells and examined for **(C, H, L)** target cell expansion and **(F, J, N)** CAR-T cell expansion. Each graph depicts automated cell counts or fluorescent areas from a single well from a single experiment.

Supplementary Figure 8 Hinge-truncated CARs display enhanced EGFR-high tumor selectivity in triple co-culture of human tumor cells, human healthy donor fibroblasts, and CAR-T cells. **(A)** Unmodified human healthy donor derived dermal fibroblasts (HDF) were placed in co-culture with equal number of mKate2-expressing SKOV3 cells. **(B)** Untransduced (mock) T cells or human T cells expressing hinge variant CAR constructs were added to HDF/SKOV3 co-cultures at equal cell number (1:1:1 ratio of HDF : SKOV3:CAR-T) and incubated with live cell monitoring using the Incucyte device; images show the state of triple co-cultures at day 6 after plating. **(C)** The fold increase of tumor cell growth at day 6 normalized to the initial plating within triple co-cultures with varying CAR-T doses was quantitated using automated cell counting. Graph depicts the average fold change in tumor cells from CAR-T cells generated from 3 independent donors +/- the standard error of the mean. **(D)** Depicts the gating strategy employed to identify CD45+ CAR-T cells, mKate2+ SKOV3 cells, and unlabelled HDF cells and examine their relative activation (CD25 expression on T cells) or viability (ZombieViolet fixability dye). Similar gating/analysis was utilized for triple co-cultures containing CAR-T cells derived from each of 3 independent blood donor samples.

Supplementary Video 1 EGFR-sdCAR-T Cells with H929 Lung tumour cells Primary sdCAR-T cells were generated from 3 independent blood donors as described in the text and placed at low density co-cultures in a 96-well plate. Cells were then monitored for growth via live fluorescence microscopy over 7 days. Images were acquired every 2 hours and assembled into a timelapse video file. Results are representative of 3 similar independent experiments using different donor cells.

Supplementary Video 2 EGFR-sdCAR-T Cells with SKOV3 Ovarian tumour cells Primary sdCAR-T cells were generated from 3 independent blood donors as described in the text and placed at low density co-cultures in a 96-well plate. Cells were then monitored for growth via live fluorescence microscopy over 7 days. Images were acquired every 2 hours and assembled into a timelapse video file. Results are representative of 3 similar independent experiments using different donor cells.

Supplementary Video 3 EGFR-sdCAR-T Cells with MCF7 Breast tumour cells Primary sdCAR-T cells were generated from 3 independent blood donors as described in the text and placed at low density co-cultures in a 96-well plate. Cells were then monitored for growth via live fluorescence microscopy over 7 days. Images were acquired every 2 hours and assembled into a timelapse video file. Results are representative of 3 similar independent experiments using different donor cells.

Supplementary Video 4 EGFR-sdCAR-T Cells with Raji lymphoma tumour cells Primary sdCAR-T cells were generated from 3 independent blood donors as described in the text and placed at low density co-cultures in a 96-well plate. Cells were then monitored for growth via live fluorescence microscopy over 7 days. Images were acquired every 2 hours and assembled into a timelapse video file. Results are representative of 3 similar independent experiments using different donor cells.

Supplementary Video 5 EGFR-sdCAR-T Cells with Ramos lymphoma tumour cells Primary sdCAR-T cells were generated from 3 independent blood donors as described in the text and placed at low density co-cultures in a 96-well plate. Cells were then monitored for growth via live fluorescence microscopy over 7 days. Images were acquired every 2 hours and assembled into a timelapse video file. Results are representative of 3 similar independent experiments using different donor cells.

Supplementary Video 6 Untransduced T Cells (Mock) with SKOV3 Ovarian tumour cells and Primary HDF cells. Primary sdCAR-T cells were generated from 3 independent blood donors as described in the text and placed at low density co-cultures in a 96-well plate. In parallel, untransduced cells were expanded and treated with a mock transduction procedure with no lentiviral vector. Cells were then monitored for growth via live fluorescence microscopy over 7 days. Images were acquired every 2 hours and assembled into a timelapse video file. Results are representative of 3 similar independent experiments using different donor cells.

Supplementary Video 7 sdAb021-45CD8h-BBz CAR-T cells with SKOV3 Ovarian tumour cells and Primary HDF cells. Primary sdCAR-T cells were generated from 3 independent blood donors as described in the text and placed at low density co-cultures in a 96-well plate. Cells were then monitored for growth via live fluorescence microscopy over 7 days. Images were acquired every 2 hours and assembled into a timelapse video file. Results are representative of 3 similar independent experiments using different donor cells.

Supplementary Video 8 sdAb021-34CD8h-BBz CAR-T cells with SKOV3 Ovarian tumour cells and Primary HDF cells. Primary sdCAR-T cells were generated from 3 independent blood donors as described in the text and placed at low density co-cultures in a 96-well plate. Cells were then monitored for growth via live fluorescence microscopy over 7 days. Images were acquired every 2 hours and assembled into a timelapse video file. Results are representative of 3 similar independent experiments using different donor cells.

Supplementary Video 9 sdAb021-22CD8h-BBz CAR-T cells with SKOV3 Ovarian tumour cells and Primary HDF cells. Primary sdCAR-T cells were generated from 3 independent blood donors as described in the text and placed at low density co-cultures in a 96-well plate. Cells were then monitored for growth via live fluorescence microscopy over 7 days. Images were acquired every 2 hours and assembled into a timelapse video file. Results are representative of 3 similar independent experiments using different donor cells.
