# Supplementary material for: Programmable Attenuation of Antigenic Sensitivity for a Nanobody-Based EGFR Chimeric Antigen Receptor Through Hinge Domain Truncation
Source: Front Immunol. 2022 Jul 22;13:864868. doi: 10.3389/fimmu.2022.864868 (PMC9354126; doi:10.3389/fimmu.2022.864868)
Supplement: Supplementary Figure 1 — Workflow for discovery and optimization of novel tumor selective sdCAR constructs: Antigen preparation, llama immunization, phage library preparation and panning, cell binding, CAR cloning, high-throughput CAR screening, and fine-tuning of CAR signaling in Jurkat cells through hinge length modulation was performed for several novel EGFR sdCAR constructs. Subsequently, confirmatory experiments were performed to test lead sdCARs in vitro and in vivo using primary human CAR-T cells. [file Presentation_1.pdf]

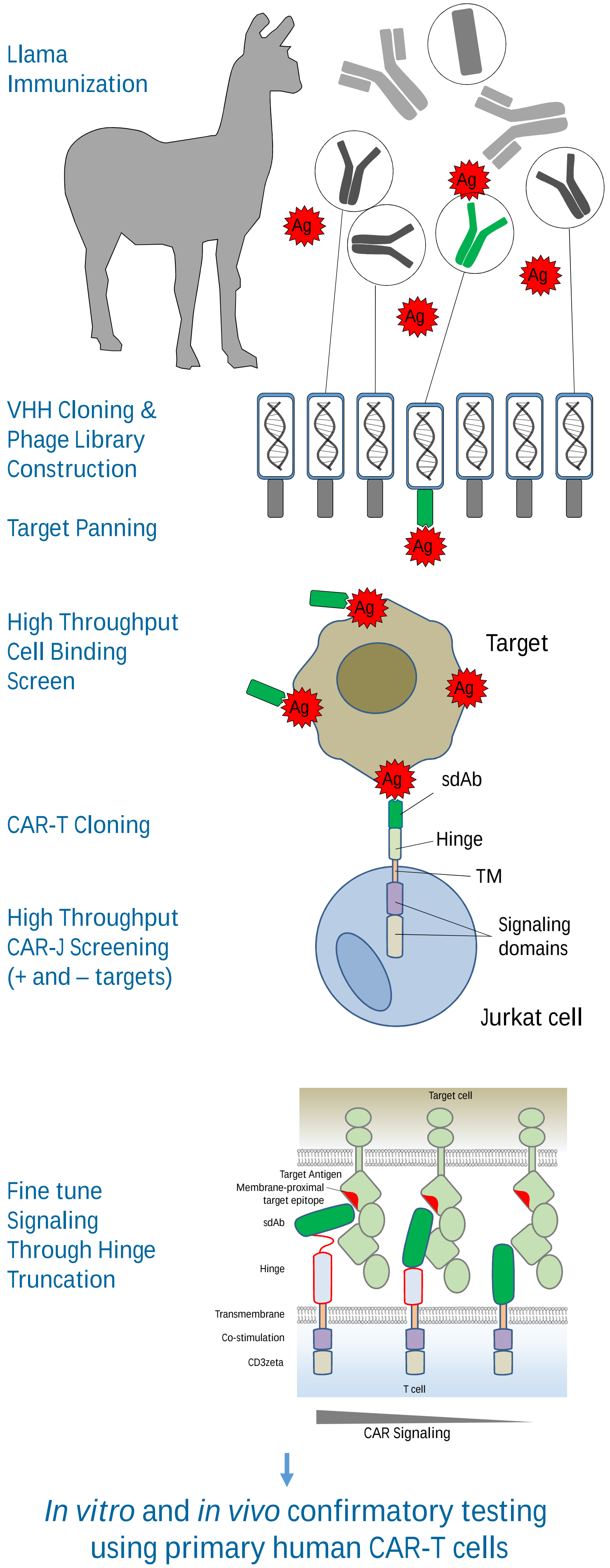

Supplementary figure 1: Workflow diagram for discovery and optimization for novel tumour selective CAR constructs

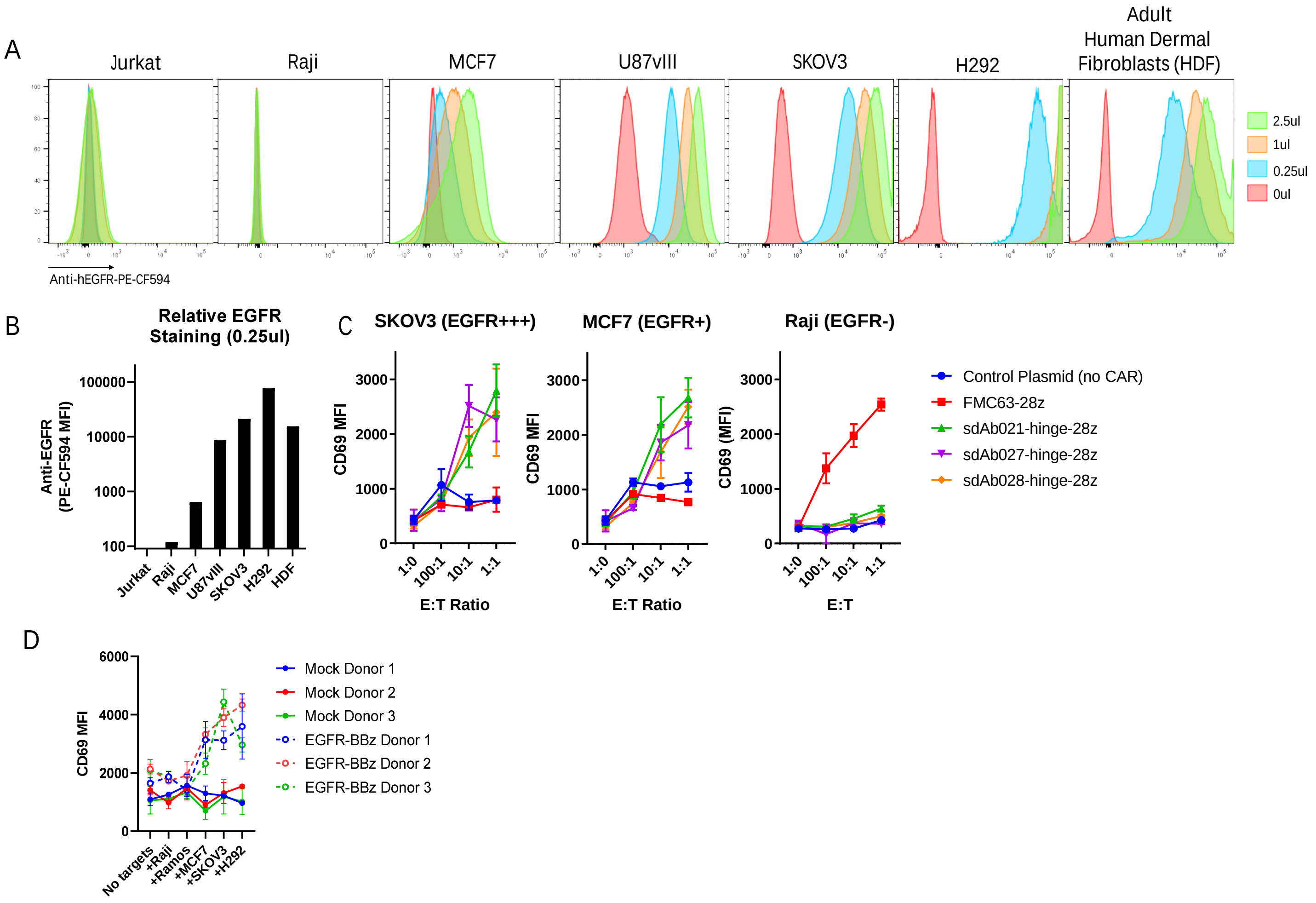

Supplemental Figure 2: EGFR-CAR-Jurkat and primary CAR-T cells can respond to EGFR-positive cells with a wide range of antigen expression

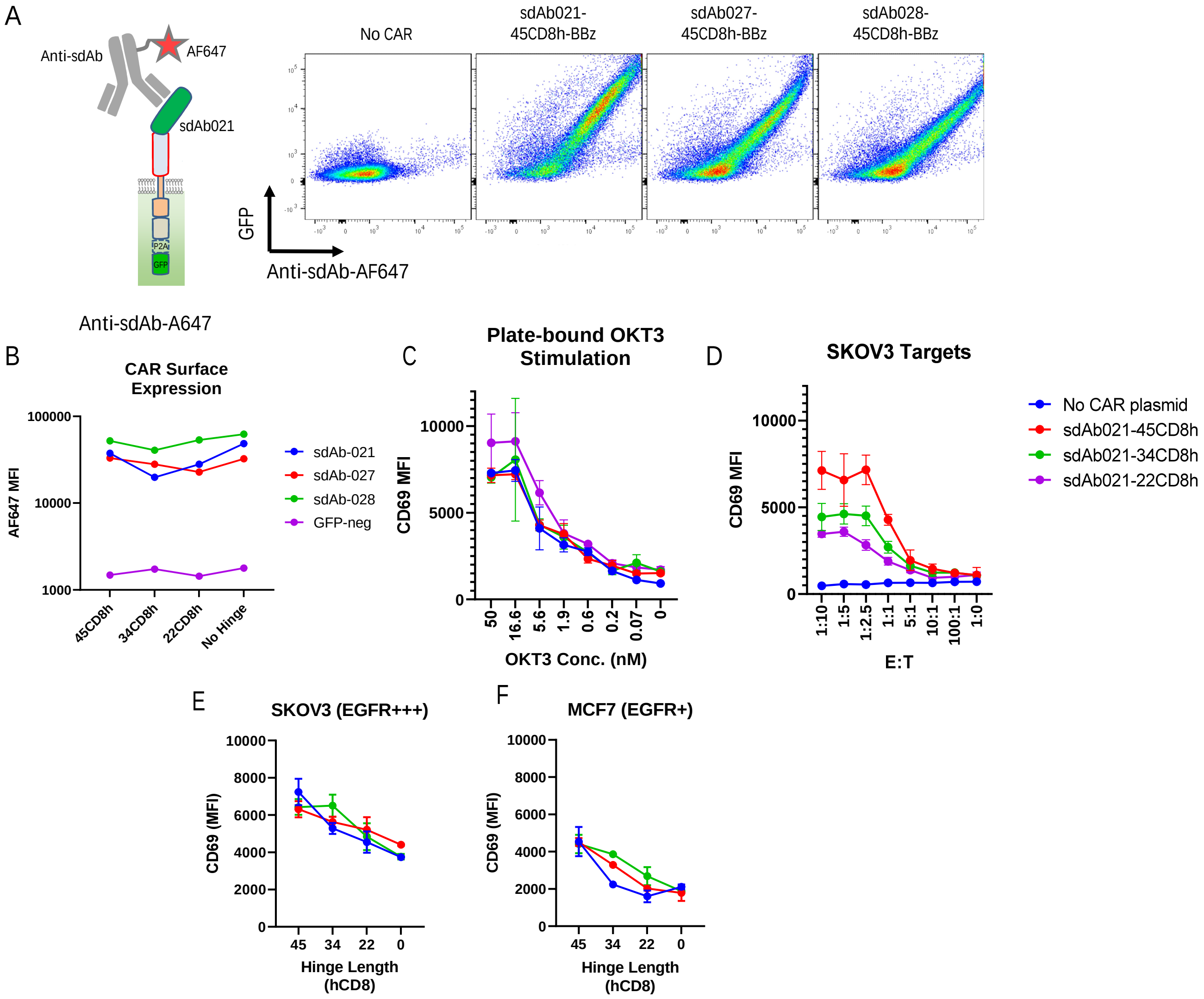

Supplemental Figure 3: CAR Surface expression is not altered with hinge domain modification

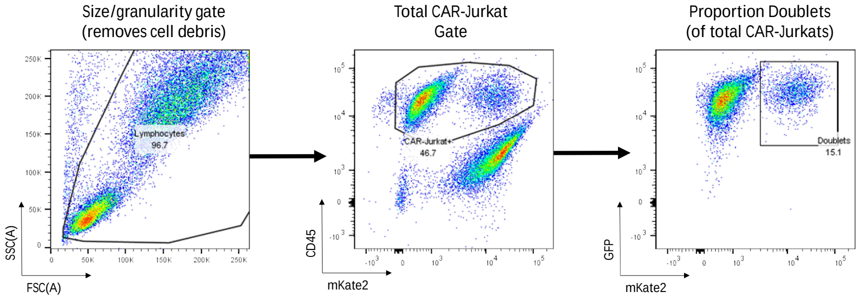

Supplemental Figure 4: Gating scheme for CAR-Jurkat target cell doublet formation assay

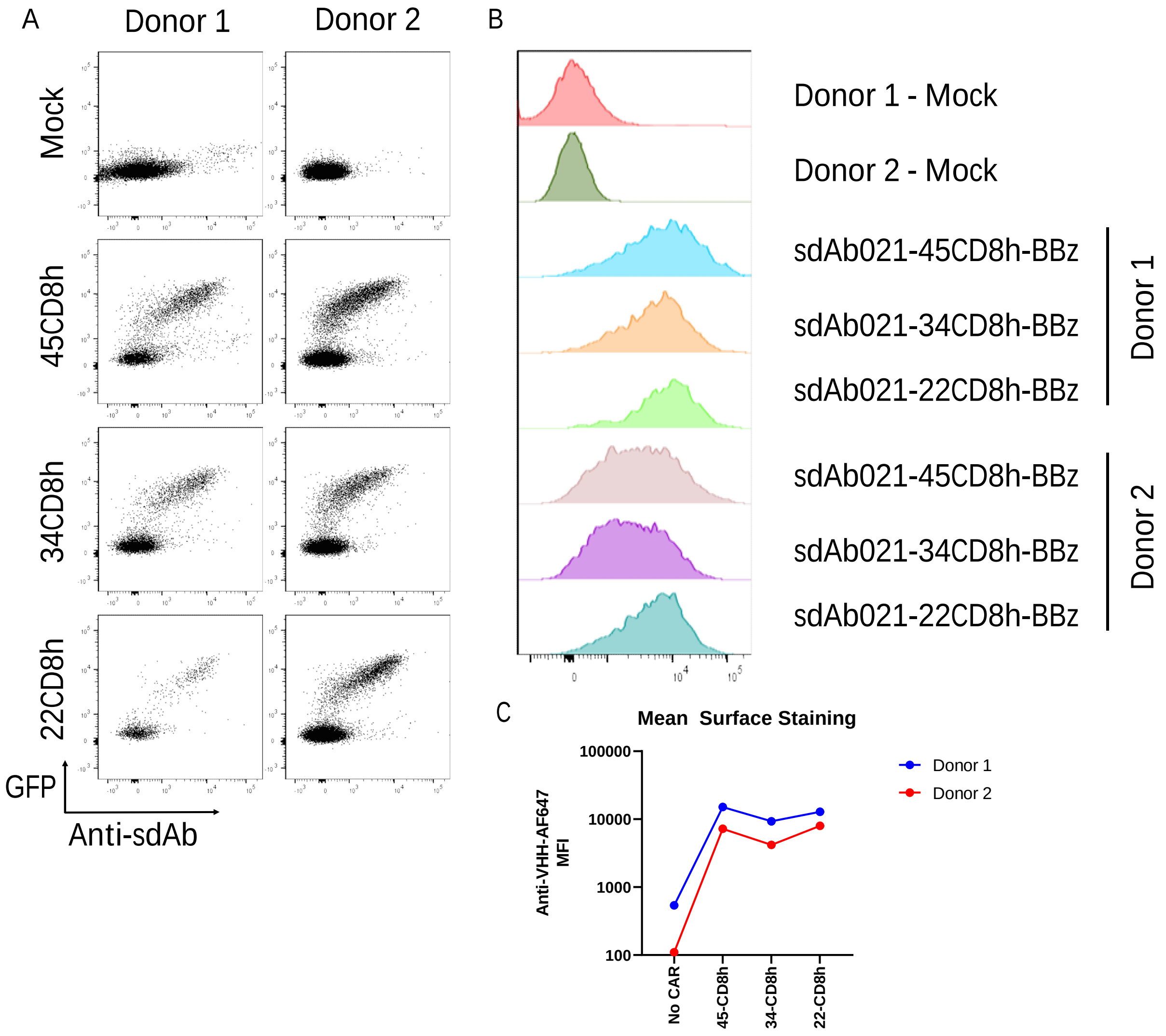

Supplemental Figure 5: CAR Surface expression in primary CAR-T cells does not correlate with hinge length

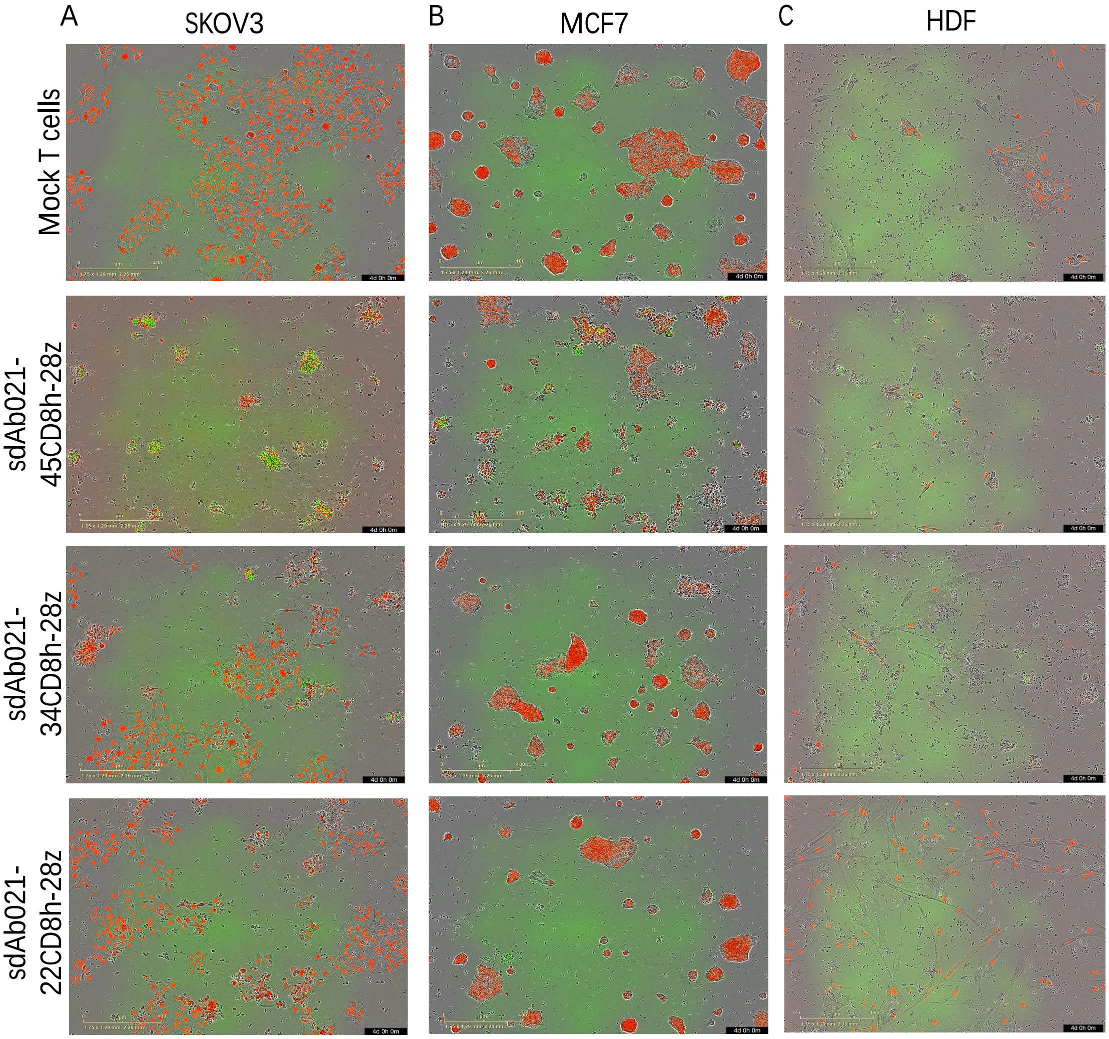

Supplemental Figure 6: Hinge truncation results in progressively reduced EGFR-sdCAR antigen responses and enhanced selectivity for EGFR-high targets

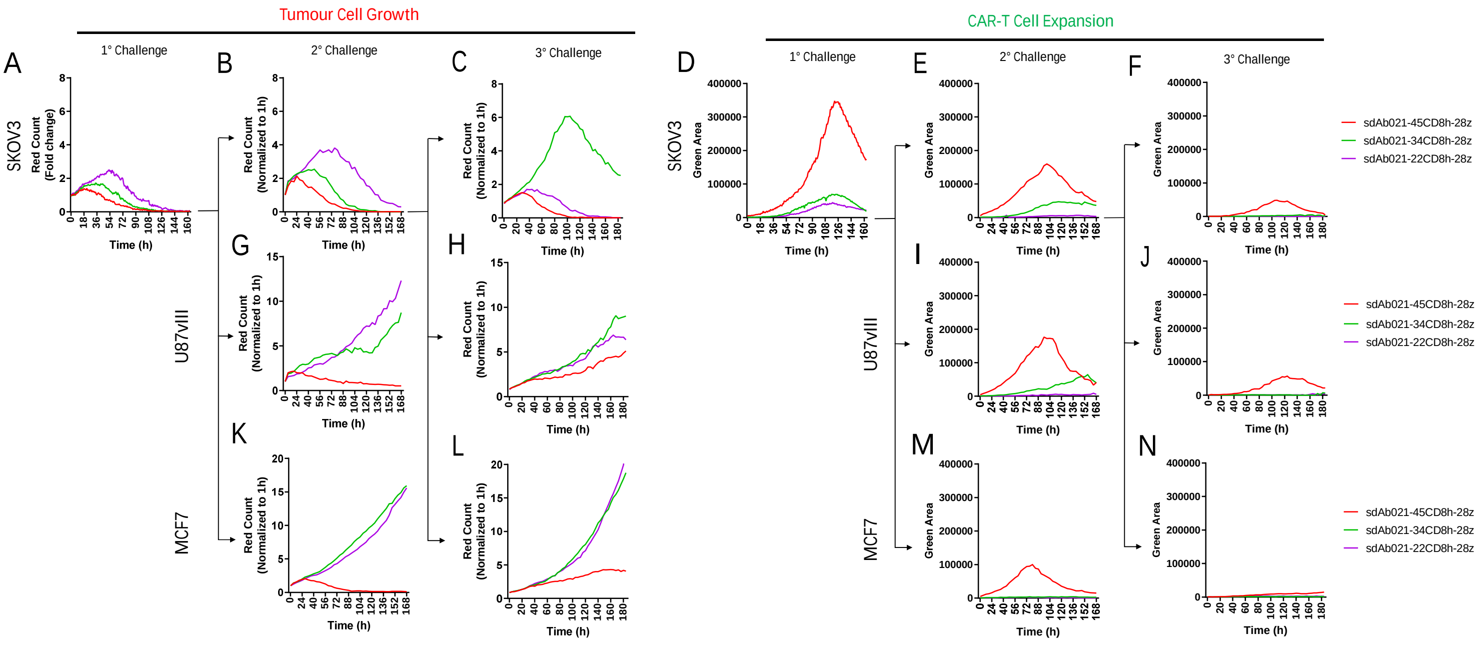

SUPPLEMENTAL Figure 7: Hinge truncated CAR-T cells maintain selectivity for target overexpressing cells following re-challenge

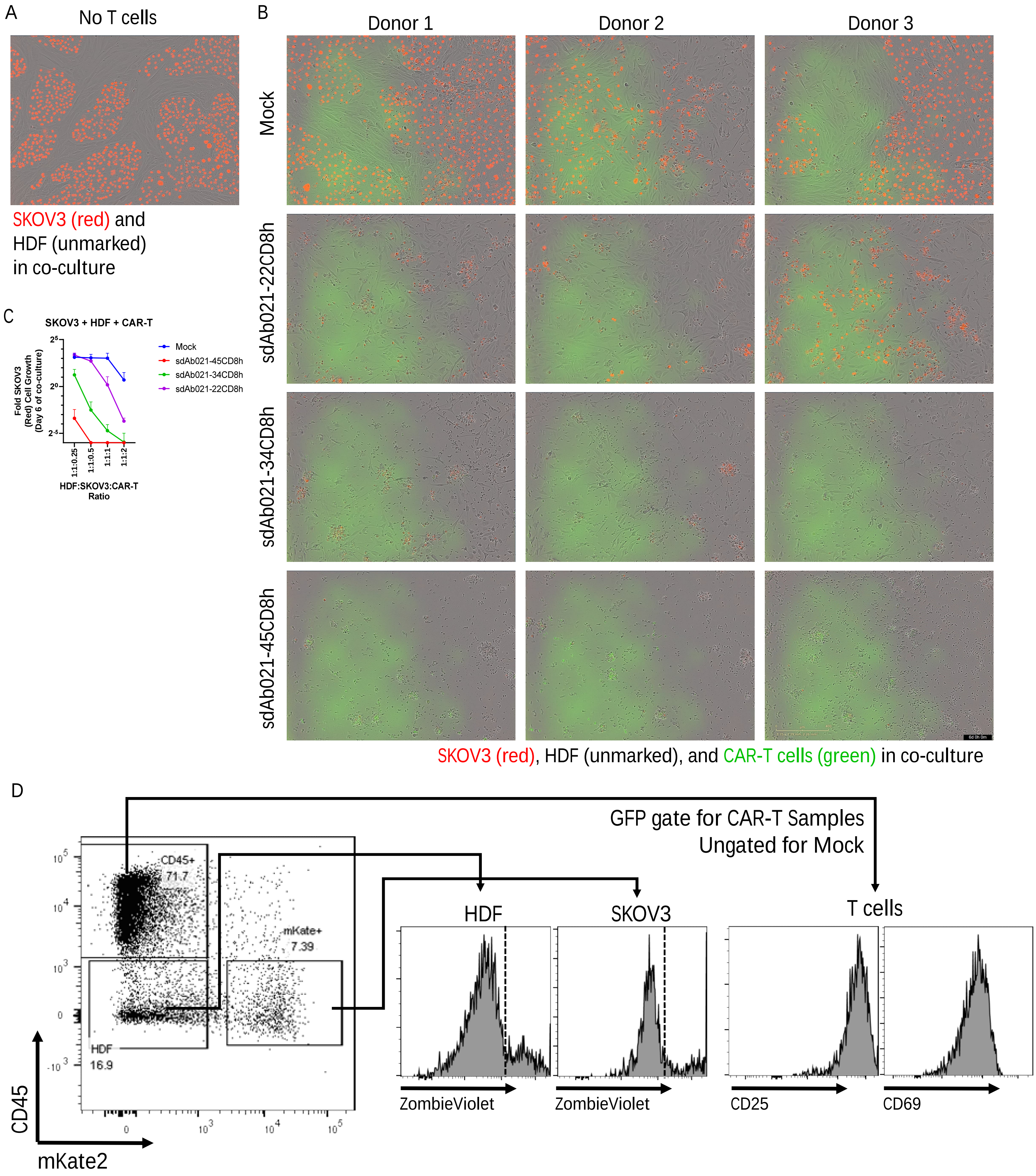

Supplemental Figure 8: Hinge truncated CAR-T cells maintain enhanced tumour selectivity in triple co-cultures with both healthy donor cells
